# A Protein-Engineered, Enhanced Yeast Display Platform for Rapid Evolution of Challenging Targets

**DOI:** 10.1021/acssynbio.1c00395

**Published:** 2021-11-22

**Authors:** Jiří Zahradník, Debabrata Dey, Shir Marciano, Lucie Kolářová, Chloé I. Charendoff, Agathe Subtil, Gideon Schreiber

**Affiliations:** † Weizmann Institute of Science, Herzl St. 234, Rehovot 7610001, Israel; ‡ Institute of Biotechnology, CAS v.v.i., Prumyslova 595, Vestec 252 50 Prague region, Czech Republic; § Institut Pasteur, Unité de Biologie cellulaire de l’infection microbienne, 25 rue du Dr Roux, Paris 75015, France

**Keywords:** protein engineering, fluorescent protein, secretory pathway, binding protein

## Abstract

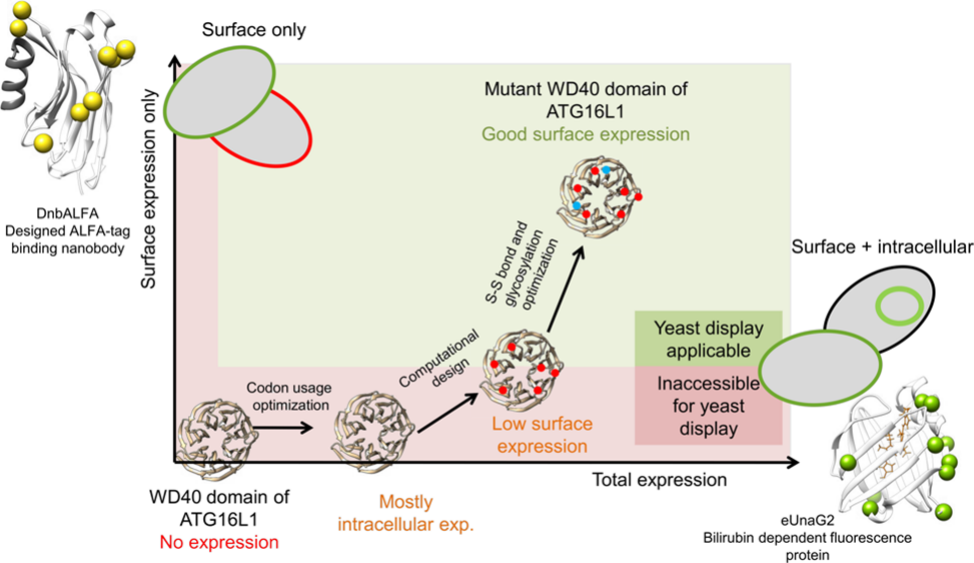

Here, we enhanced the popular yeast display method by multiple rounds of DNA and protein engineering. We introduced surface exposure-tailored reporters, eUnaG2 and DnbALFA, creating a new platform of C and N terminal fusion vectors. The optimization of eUnaG2 resulted in five times brighter fluorescence and 10 °C increased thermostability than UnaG. The optimized DnbALFA has 10-fold the level of expression of the starting protein. Following this, different plasmids were developed to create a complex platform allowing a broad range of protein expression organizations and labeling strategies. Our platform showed up to five times better separation between nonexpressing and expressing cells compared with traditional pCTcon2 and c-myc labeling, allowing for fewer rounds of selection and achieving higher binding affinities. Testing 16 different proteins, the enhanced system showed consistently stronger expression signals over c-myc labeling. In addition to gains in simplicity, speed, and cost-effectiveness, new applications were introduced to monitor protein surface exposure and protein retention in the secretion pathway that enabled successful protein engineering of hard-to-express proteins. As an example, we show how we optimized the WD40 domain of the ATG16L1 protein for yeast surface and soluble bacterial expression, starting from a nonexpressing protein. As a second example, we show how using the here-presented enhanced yeast display method we rapidly selected high-affinity binders toward two protein targets, demonstrating the simplicity of generating new protein–protein interactions. While the methodological changes are incremental, it results in a qualitative enhancement in the applicability of yeast display for many applications.

## Introduction

Macromolecular interactions are a driving force for most processes in life. Proteins bind fast and specific even in the crowded environment of the cell, transferring signals, building complexes, transport cargo, and much more. This happens in an incredible range of concentrations, from millimolar to femtomolar. The generation of novel, specific interactions has been a major goal of protein engineers from the beginning. For example, the generation of novel antibodies binding specific targets has revolutionized medicine, as acknowledged by the 2018 Nobel prize in chemistry, which was awarded for the development of the phage display method for in vitro evolution of antibodies to specifically bind any given target. Phage display was the first of many other in vitro evolution methods since devised. In 1993, the pioneering work of Schreuder and colleagues^1^ first described yeast display, which over time became the most widely used method for directed protein evolution. Similarly to other display methods, its principle is based on cycles of naïve protein library exposure, selection, and enrichment of yeast clones with desired properties. Yeast display has proven to be an effective method for developing,^2,3^ improving,^4^ and altering activities^5,6^ of proteins for research, therapeutic, and biotechnology applications. The unprecedented power of the technique, together with its relative ease of use and reasonable cost, has made it popular in many laboratories around the world. The use of *Saccharomyces cerevisiae* and its homologous recombination machinery reduces the need for laborious DNA library preparations, with only DNA fragments being needed.^7,8^ Coupling of the genotype–phenotype association with high-throughput single-cell analysis on a fluorescent activated cell sorter (FACS) offers a simple and efficient screening process, with a low risk of false-positive results.^9^

The most popular yeast display setup is based on the yeast mating factor agglutinin A protein, which is composed of two independent domains: the A-agglutinin GPI-anchored subunit (Aga1p) and the adhesion subunit (Aga2p).^1^ The subunit interaction is mediated by two disulfide bridges, and the protein of interest is fused to the plasmid-encoded Aga2p subunit.^10^ Both C and N terminal fusions with Aga2p were used for successful display on the yeast surface.^4,11^ Multiple Aga2p fusion partners and their libraries were screened and tailored to fulfill a plethora of tasks such as affinity reagent development,^12−14^ substrate specificity modulation,^15^ protein stability engineering,^16^ and also for directed evolution of enzymes.^17,18^ Still, despite all of these developments, yeast display selection of optimal binders is challenging and time-consuming. Therefore, we looked for substantial simplification of the current yeast surface display methodology that will also improve handling of difficult-to-express proteins.

## Results

### Part 1: Development of the Enhanced Yeast Display Platform

#### Yeast Display Plasmid Optimization

The most frequently used plasmid for yeast display, pCTcon2, was developed more than 15 years ago,^19^ before advanced DNA manipulation technologies such as restriction-free cloning^20^ were developed. Therefore, the pCTcon2 is rich with unnecessary sequences incorporated during its initial assembly. To simplify the work with pCTcon2, we modified its backbone to create a new plasmid system. First, we replaced the *AmpR* gene with *KanR* coding for aminoglycoside-3′-phosphotransferase, as kanamycin is more stable over time.^3,21^ Next, we removed unnecessary sequences, like T7, T3 promoter regions, F1 origin of replication, lac operator, and promoter fragments using three-component assembly by restriction-free cloning.^22^ The mutual position of functional elements was kept the same as in pCTcon2. The resulting vector was designated pJYD (plasmid J-series yeast display), and its full-length sequence was verified. pJYD is 1301 bp shorter than the parental vector pCTcon2 (6456 bp). Full-length sequences are available via Addgene repository (Table 1).

**Table 1:** Summary of Antibody Labeling-Free Yeast Display Platform Plasmids

Addgene ID	plasmid name	N terminus MCS/reporter site	linker	anchor	linker	C terminus MCS/reporter site	C-terminal tag
162,450	**pJYDN**	MCS-negative	NGL linker	Aga2p	HA tag, Myc tag	eUnaG2	
162,451	**pJYDNp**	MCS-positive	NGL linker	Aga2p	HA tag, Myc tag	eUnaG2	
162,452	**pJYDNg**	MCS-negative	2G linker	Aga2p	HA tag, Myc tag	eUnaG2	
162,453	**pJYDNgp**	MCS-positive	2G linker	Aga2p	HA tag, Myc tag	eUnaG2	
162,454	**pJYDN2**	MCS-negative	NGL linker	Aga2p	HA tag, Myc tag	DnbALFA	
162,455	**pJYDN2p**	MCS-positive	NGL linker	Aga2p	HA tag, Myc tag	DnbALFA	
162,456	**pJYDN3**	ALFA-tag, MCS-negative	NGL linker	Aga2p	HA tag, Myc tag	eUnaG2	
162,457	**pJYDN3p**	ALFA-tag, MCS-positive		Aga2p	HA tag, Myc tag	eUnaG2	
162,458	**pJYDC1**	eUnaG2		Aga2p	HA tag	MCS–HDEL	Myc-tag
162,459	**pJYDC2**	eUnaG2		Aga2p	HA tag	MCS–HDEL	ALFA-tag
162,460	**pJYDC3**	DnbALFA	NGL linker	Aga2p	HA tag	MCS-HDEL	Myc-tag
accessory plasmids							
	pET28bdSUMO-CyPet-ALFA				pET28bdSUMO-CyPet-DnbALFA		
	pET28bdSUMO-mNeon-ALFA				pET28bdSUMO-mNeon-DnbALFA		
	pET28bdSUMO-eUnaG2-ALFA				pET28bdSUMO-eUnaG2-DnbALFA		
	pET28bdSUMO-YPet-ALFA				pET28bdSUMO-YPet-DnbALFA		
	pET28bdSUMO-mCardinal-ALFA				pET28bdSUMO-mCardinal-DnbALFA		

#### Screening for Expression Reporters

We tested a broad range of four green and five far-red fluorescent proteins and two nanobodies of different sizes and properties. The two colors were chosen to fit the most popular FACS fluorescence setup, corresponding to green and red channels FL1 and FL4, respectively, while allowing tests such as propidium iodide viability staining.^23^ We examined the FACS expression characteristics of green fluorescent proteins mNeonGreen,^24^ yeast-enhanced green fluorescent protein (yeGFP),^25^ bilirubin-inducible fluorescent protein UnaG,^26^ FMN-inducible fluorescent protein iLOV,^27^ biliverdin-binding far-red fluorescent proteins dFP-mini,^28^ GAF-FP,^29^ TDsmURFP^30^ with two Y56R mutations,^31^ IFP1.4,^32^ and miRFP670nano.^33^ In addition, we tested two peptide tags recognizing nanobodies: BC2 nanobody (nbBC2)^34^ and ALFA nanobody (nbALFA).^35^ The codon-optimized genes^36^ were cloned into the pJYD plasmid under the control of the GAL1 promoter in two different positions to obtain plasmids with protein expression at different localizations—cytoplasmic expression replacing the Aga2p including its signal peptide and cell surface expression, fused with Aga2p. Details of the cloned positions are given in Supporting Information text 1. When testing UnaG protein, the expression media were supplemented with 2.5 nM bilirubin to obtain the reporter in its fluorescent form because *S. cerevisiae* EBY do not produce bilirubin naturally. Bilirubin itself is nonfluorescent and therefore does not cause any false-positive signal. Media for the cultivation of biliverdin-binding far-red fluorescent proteins were supplemented with 5 nM biliverdin.

**Figure 1:**
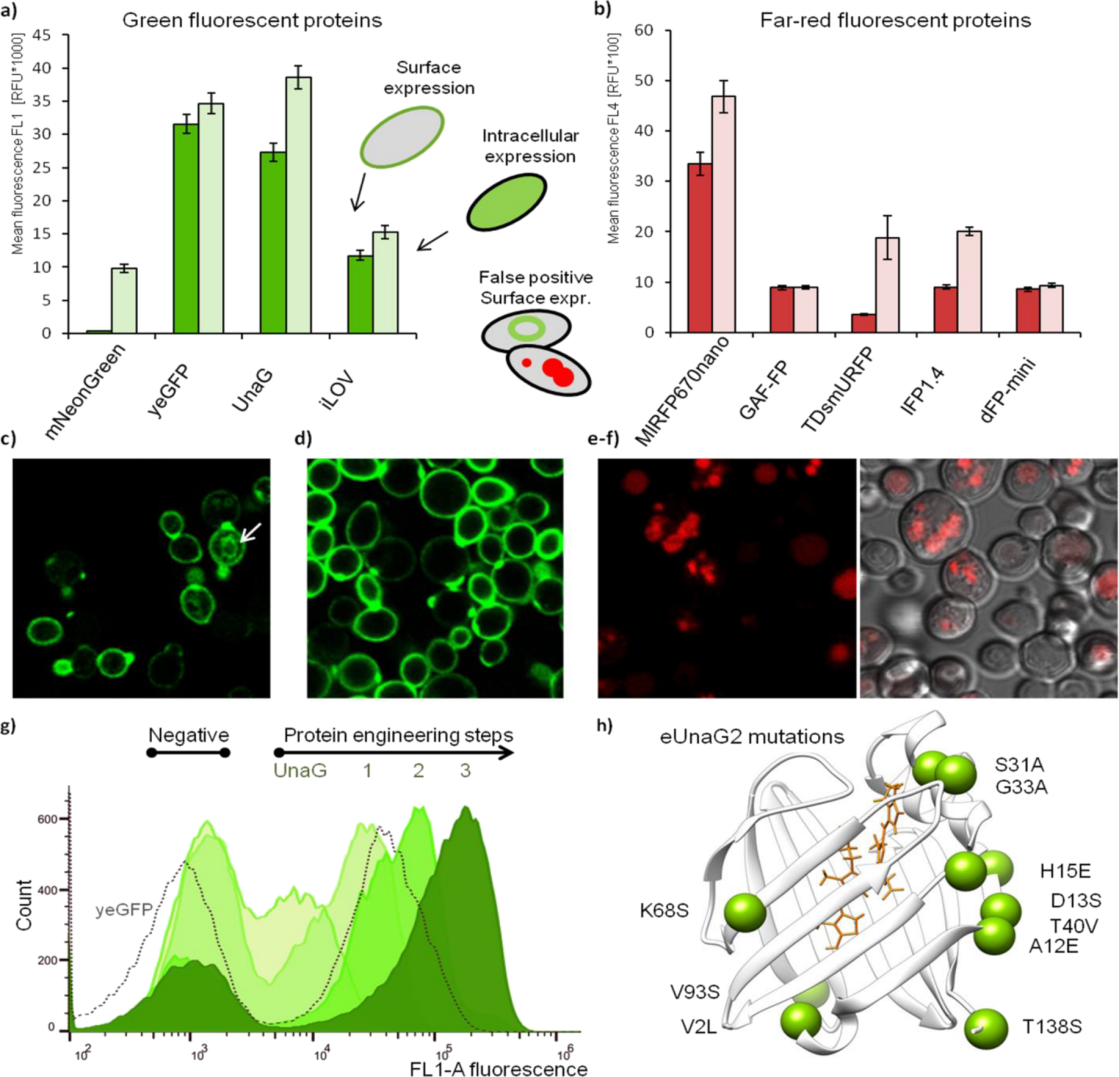
Evaluation and engineering of fluorescent proteins for optimal yeast surface exposure. Comparison of cytometry-assessed mean fluorescence intensities for (a) green and (b) far-red fluorescent proteins between Aga2p fusion on the cell surface (rich color) and intracellular expression (faint color). (c–f) Microscopy images of *S. cerevisiae* EBY100 cells expressing (c) yeast-enhanced yeGFP; the false-positive signal from yeast endoplasmic reticulum is marked by the white arrow. (d) UnaG bilirubin-dependent fluorescent protein and (e and f) miRF670nano protein. In contrast to yeGFP and UnaG, the miRF670nano protein fused to the C-terminus of Aga2p was not detected on the yeast surface. (g) Flow cytometry histograms showing the green fluorescence signal (FL1 channel) distribution among cell populations during the subsequent protein engineering steps of UnaG. The dotted line shows yeGFP protein for intensity and distribution comparison. (h) Mutations introduced during the eUnaG2 protein engineering depicted in the 3D structure of UnaG protein (PDB id: 4i3b).

We compared fluorescence intensity differences after 16 h of expression at 20 °C by flow cytometry. Results were further validated using fluorescence microscopy (Figure 1a–c). The presence of a characteristic inner ring for endoplasmic reticulum and Golgi or fluorescence foci suggesting the presence of fluorescent proteins in vacuoles was analyzed to uncover impaired reporter processing to the cell membrane.^37^

Our cytometry results showed the highest fluorescence intensities of surface-exposed protein for the yeGFP construct, while UnaG showed higher cytoplasmic fluorescence levels. Validation by microscopy of yeGFP showed a higher proportion of fluorescent signals emitted from the endomembrane than that observed for UnaG (Figure 1c,d). Folding of fluorescent proteins inside the endoplasmic reticulum or other endomembrane compartments can be a source of false-positive signals in yeast surface display and should be avoided or decreased. Based on these results, its smaller size, and the ability to control the fluorescence by the addition of bilirubin to the cultivation media, we used the UnaG protein for further tailoring its properties to best fit our yeast display setup.

Among far-red fluorescent proteins, only miRF670nano showed a satisfactory fluorescence signal on flow cytometry (Figure 1b). The microscope showed most of the recorded signals coming from inside the cells (Figure 1e,f). By the appearance of miRF670nano inside fluorescence foci, we hypothesized its localization to be mainly vacuolar. Therefore, we decided not to continue with biliverdin-dependent far-red fluorescent proteins and instead focused on nanobodies.

Both nbALFA and nbBC2 nanobodies showed high expression levels on the yeast surface when expressed as C-terminal fusions to Aga2p in the pJYD vector and labeled with c-myc antibody-based labeling. The FACS signal for ALFA-tag binding nanobody showed a 24% better signal than the BC2 tag binding nanobody (Figure S1a). Based on the more robust expression at the yeast surface and higher affinity to its cognate tag, we decided to continue using nbALFA.^35^

Next, we analyzed the ability of our reporter genes to be produced in an active, fluorescent form during the yeast cultivation in expression media. We tested whether the addition of bilirubin to the media will be sufficient for the UnaG fluorescent protein to be in its holo form without affecting yeast growth. In the range of concentrations tested (100 μM–1 pM), we did not observe significant growth inhibition. Fluorescence saturation was observed with >200 pM bilirubin. Optimal labeling of yeast was achieved using 1 nM bilirubin in the expression media added directly before the cultivation from frozen, DMSO diluted stock. This concentration led to a slight media color change. The expression labeling using nbALFA during the cultivation was tested with different concentrations of ALFA-tagged mNeonGreen, produced in *E. coli* BL21. The stability of the ALFA-tagged mNeonGreen in conditioned media after 48 h of expression was determined by measuring the fluorescence on a plate reader (Figure S2).

#### Engineering UnaG for Efficient Cell Surface Expression and Brighter Green Fluorescence

An ideal expression reporter for yeast display should have bright fluorescence and a low level of the false-positive signal from inside the cell. Yeh et al.^38^ used yeast surface display to increase UnaG fluorescence, generating the eUnaG protein (enhanced UnaG). One single-point mutation in eUnaG, V2L, led to an increase in thermal stability by almost 6 °C and doubling of the fluorescence intensity signal, suggesting that higher UnaG stability leads to increased fluorescence intensity. To further improve the protein’s stability, we combined computational and experimental procedures. Multiple stabilizing mutations were predicted by the consensual design of all available structures using the Pross web-server.^39,40^ Because Pross was not designed to optimize secreted/surface-exposed proteins, we used the PROSS-suggested mutations as a starting point for random incorporation and selection, rather than testing suggested protein variants. A mutation library with more than 10^7^ clones was created from the 13 in silico predicted mutations using the transfer-polymerase chain reaction (TP-PCR) technique^41^ and a set of mutagenic primers with predicted mutations. Cells associated with stronger fluorescence intensities were isolated by three rounds of FACS sorting. Stronger fluorescence was verified for the isolated single clones. The brightest isolated clone eUnaG1 showed a significantly higher fluorescent signal when using FACS than the parental variant (Figure 1g, engineering step 1). eUnaG1 was further improved by incorporating additional four mutations found in other selected colonies (Figure 1g, engineering step 2). Finally, our inspection of the crystal structure PDB ID 4i3b showed a solvent-exposed hydrophobic patch formed between residues V89, V93, V98, V100, and V111. Because surface polarity is a critical parameter influencing expression,^42^ we designed and tested the gain of *N*-glycosylation mutations (V93S and E107N) to rescind this patch. The gain of *N*-glycosylation was mediated by mutations leading to a new N-X-S/T surface-exposed motif.^43^ The V93S mutation led to a further doubling of fluorescence intensity (Figure 1g, engineering step 3). Altogether, we introduced 10 mutations in eUnaG2 (Figure 1h), which results in a 5-fold increase in its fluorescence intensity as measured by FACS. eUnaG2 expressed in *E. coli* BL21 cells showed an increased bilirubin binding and thermal stability relative to UnaG of 10 °C (Figure S1b, c).

#### Engineering the ALFA-Tag Binding Nanobody for Efficient Multicolor Fluorescence Labeling

The engineering of the ALFA-tag binding nanobody for increased thermal stability was a more challenging task, as computational tools for ΔΔG predictions of antibodies have higher false-positive rates.^39,44^ Ten mutations were predicted and tested one by one (Figure S1d). The proteins were expressed in *E.coli* and purified, and their melting temperatures were measured using the nanoDSF Prometheus NT.48. We identified eight mutations with *T*_m_ values ranging from 54 °C (wild type) to 55.3 °C (best mutant Q69K) which is only 1.3 °C higher than the wild type. Combining them (see Figure S1d) leads to an increase of 6 °C in thermal stability. In a subsequent, second round of protein engineering, we screened two *N*-glycosylation gaining mutations (G17N and T25N) and their influence on the expression of the nanobody. Both of them slightly enhanced the protein expression, although it decreased the thermal stability of the protein by 4 °C. The melting temperature of glycosylated proteins was measured directly on yeast using the interaction with purified ALFA-tagged mNeonGreen.^16^ Among all tested mutations, 10 mutations had positive effects on the recorded cytometry signals and were combined in the final construct of the protein, termed Designed ALFA-tag binding nanobody (DnbALFA). Figure S1e shows the development of the cytometry signal along with the protein-engineering steps. Measuring the binding affinity of DnbALFA and nbALFA toward ALFA-mNeonGreen showed a 2-fold reduction of the former (60 versus 25 pM respectively, Figure S1f). Despite the slight decrease in binding affinity, the gain in protein expression is much higher both on the yeast cell surface and in *E.coli* BL21 (DE3), showing a 10-fold increase in yield of the soluble designed protein (Figure S1g).

#### Yeast Display Platform Design and Engineering

In the next step, we incorporated eUnaG2 or DnbALFA reporter proteins either in the N or C-terminal vector, introduced the multicloning sites (MCSs), and tested signal peptides and linkers to achieve optimal surface expression. Different plasmids were developed to create a complex platform allowing a broad range of protein expression organizations and labeling strategies. Our aim here was to create an enhanced yeast display system with advantages in speed, variability of expression, and selection over the standard anti-myc antibody system. However, this variability requires careful experimental design. The following chapters are describing the plasmids’ development and their limitations. Further details are in Supporting information texts 2 and 3.

#### Construction of N-Terminal Vectors

**Figure 2:**
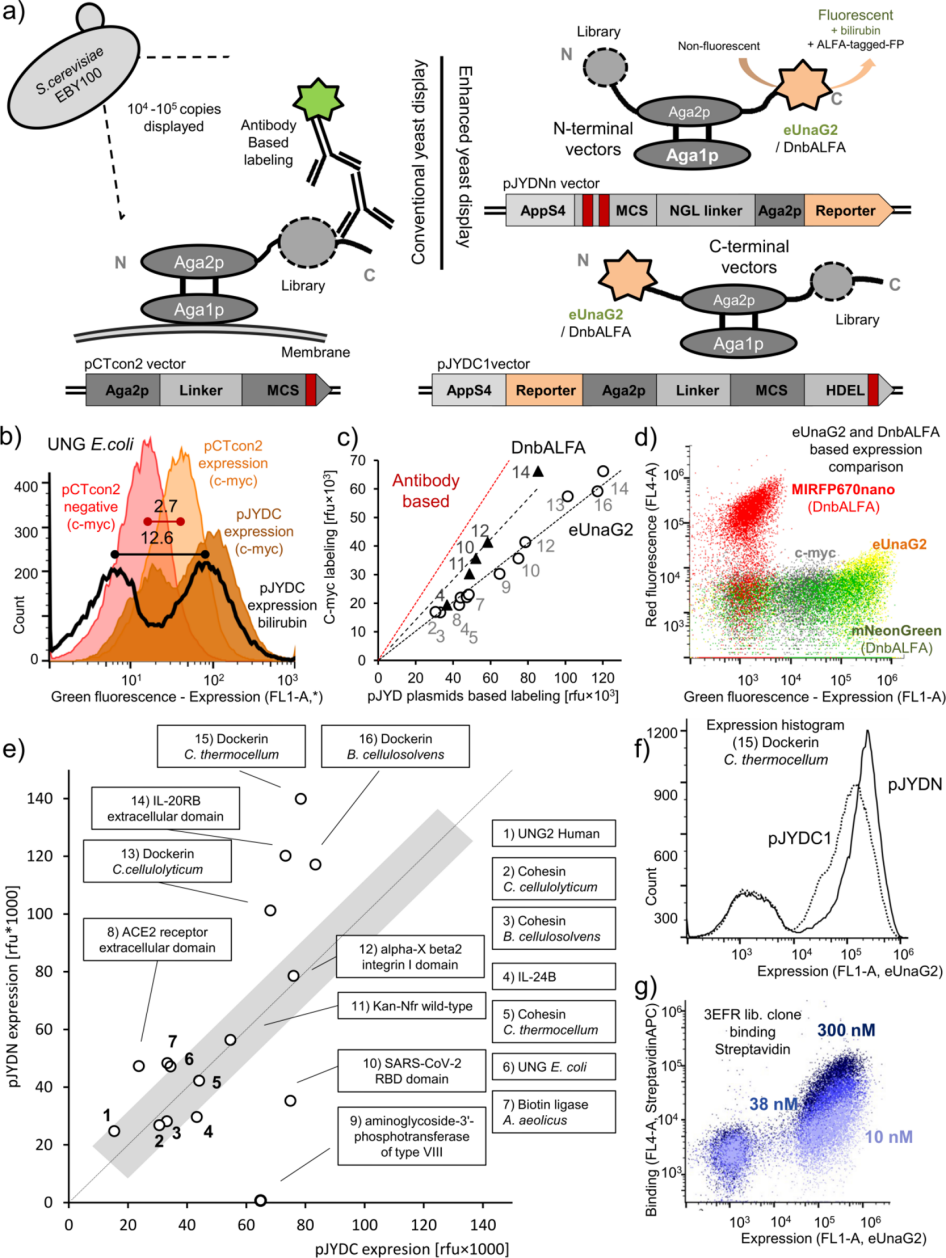
Comparison between traditional and enhanced yeast display**. (**a) Schematic comparison of traditional yeast display based on mating agglutinin and enhanced yeast display. (b) Comparison of UNG protein from *E. coli* expression in the original pCTcon2 plasmid and pJYDC plasmid labeled by traditional c-myc tag labeling (red and orange histograms) and eUnaG2 (black line histogram). The separation between negative and positive populations is highlighted by horizontal lines and accompanied by the signal ratio. (c) Comparison of expression labeling intensities between traditional antibody-based c-myc labeling (pCTcon2) and the here-engineered eUnaG2 (circles, pJYDC1) and DnbALFA (triangles, pJYDC3) alternatives for proteins with minimal retention inside cells. The numbers correspond to those in panel d, giving the identities of the proteins. (d) FACS fluorescence dot plot signal comparison between eUnaG2 reporter (yellow, pJYDNp), DnbALFA coupled with ALFA-mNeonGreen (green, pJYDN2p), or ALFA-miRFP670nano protein (red), and traditional anti-c-myc (gray, pJYDNp). The eUnaG2 protein excitation maximum is at 498 nM, and the emission maximum is 527 nM, which caused a small signal spillover into the red channel as evident at high signal intensities. A routine compensation procedure can be applied for signal correction. (e) Differences in yeast surface expression between N (pJYDN) and C (pJYDC) terminal protein fusions with Aga2p among 16 tested proteins. The gray area highlights equal expression in both vectors (± 7500 rfu). (f) Overlay of expression histograms for dockerin from *C. thermocellum* (no. 15) expressed in pJYDC1 (C-terminal fusion with Aga2p) and pJYDN (N-terminal fusion). The comparison demonstrates higher expression and uniformity for dockerin fused with Aga2p at the N-terminus. (g) Binding signal recorded together with eUnaG2 expression labeling. Stacked dot plots were acquired after incubation of 3EFR-Cfr-anti-Streptavidin-APC with 10, 38, and 300 nM Streptavidin-APC for 1 h.

In the N-terminal fusion organization, the selected protein is bound N-terminal, between the signal peptide and Aga2p, which is opposite to its location in the traditional pCTcon2 construct (Figure 2a).^4,11^ This construct has the advantage of the presence of a reporter gene at the C terminus, avoiding incorporation of stop-codons into the mutated protein.

To develop our plasmid system, we tested the impact of different signal peptides and linkers. The best performance was observed for shortened (AA 1–23) appS4 secretory leader^45^ (Figure 2a) and linkers bearing *N*-glycosylations. Details are given in Supporting information text 2 and Figure S3. Engineered reporters were incorporated at the C terminus between *Bam*HI and *Xho*I restriction sites. Plasmids containing eUnaG2 and DnbALFA were designed pJYDNp and pJYDN2p, respectively (Table 1). These plasmids do not contain stop-codons, are expressed in appropriate galactose-containing media, and were used as expression controls. Finally, we created additional plasmids with two consecutive stop-codons being introduced into the MCS (Figure 2a). This comes to avoid the possibility that the empty plasmid will give rise to a fluorescent signal. These plasmids are referred to as negative plasmids (pJYDNn, pJYDN2n) and can be used either as a negative control or template for plasmid cleavage and subsequent homologous recombination without the risk of false-positive colonies with empty plasmids.

#### Construction of C-Terminal Vectors

The C-terminal vectors resemble parental pCTcon2 organization with the protein of interest or library being fused to the C-terminus of Aga2p and reporters at the N-terminus of Aga2p (Figure 2a). This expression organization requires appropriate experimental design and controls because of the risk of a false-positive signal given by truncated clones.

We used the above described N-terminal testing vector and cloned eUnaG2 at the N-terminus of the Aga2p and restored the original pCTcon2 MCS site at the C-terminus (Figure 2a). The control experiment showed high eUnaG2 fluorescence in an empty vector. To limit this empty plasmid expression, we introduced *S. cerevisiae* endoplasmic reticulum-targeting peptide HDEL^46^ at the C-terminus of the MCS sequence between the *Nde*I and *BamH*I sites. Indeed, we confirmed using microscopy that the eUnaG2/DnbALFA-Aga2p-HDEL construct was predominantly retained in the endoplasmic reticulum (Figure S4a–c) with its fluorescence being reduced by almost 5-fold compared to the plasmid without a retention signal. This reduces the false-positive signal from an empty plasmid emerging in library construction. To ensure that we detect only full-length constructs at the yeast surface, the C-terminal myc-tag was retained in the plasmid, and we also created vectors with ALFA-tag to enable traditional labeling (pJYDC2).

#### Plasmid Construction

Based on our optimized N and C terminal plasmids and engineered reporter proteins, we constructed multiple yeast display vectors with different combinations of functional elements. The different plasmids and their functional element organizations are schematically shown in Table 1. All plasmids allow for labeling-free and traditional labeling procedures offering thus maximal versatility in the experimental design and monitoring of protein processing.

All yeast display plasmids were deposited in the Addgene plasmid repository, and their corresponding ID numbers are shown in Table 1. Table 1 also shows the plasmids constructed to complement our yeast display vectors with vectors for the production of fluorescent proteins: ALFA-tagged fluorescent proteins and fluorescent proteins fused with DnbALFA. The expression vectors are based on pET28bdSUMO vector^40^ and enable bdSUMO protease single-step, on-column cleavage-based purifications.^47^ An example of proteins purified by this single-step purification process is shown in Figure S5. Both ALFA-tagged proteins and DnbALFA fusions do not require further purification steps and can be used directly for cocultivation labeling. The use of different plasmids and additional details are given in the step-by-step protocol for enhanced yeast display (Supporting information text 3).

#### Examining Protein Expression Using the Enhanced Yeast Display Platform

To test the applicability and performance of our yeast display system, we initially compared the expression of the *E. coli* UNG protein in the original pCTcon2 plasmid and pJYDC plasmid (Figure 2b). Expression from both plasmids was labeled with anti-c-myc antibody-based labeling with secondary antibody conjugated to Alexa Fluor 488.^9^ The pJYDC showed higher expression and better separation between the negative and positive populations (Figure 2b). This shows that the new plasmid pJYDC significantly improves UNG protein surface expression by combining optimized plasmid components and the presence of the yeast display-tailored eUnaG2 reporter. Finally, we compared previously measured expressions with the pJYDC-UNG (*E.coli*) measured by the eUnaG2 reporter (Figure 2b, black histogram) and obtained 4.7 times better separation between the signal of nonexpressing and expressing cells, compared to the pCTcon2. This results in much higher separation of expressing/binding clones per cycle of selection. Next, we analyzed the expression of 16 well-expressed proteins using traditional anti-c-myc antibody-based labeling (pCTcon2 plasmid), intrinsic eUnaG2 fluorescent signal (pJYDC1 plasmid), and DnbALFA with cocultivation labeling with ALFA-mNeonGreen (pJYDC3 plasmid). Our results show a very tight correlation in the strength of the fluorescence signal between the three systems for a subset of tested proteins. This suggests that the expression signal is proportional among the plasmids and proteins with the absolute fluorescence intensities being the brightest for eUnaG2 (double of c-myc) followed by DnbALFA—mNeonGreen labeling (50% increase over c-myc), Figure 2c. A comparison between the pJYDNp, positive control plasmid expression (eUnaG2 reporter protein in yellow), and pJYDN2p plasmid expressing DnbALFA-tagged mNeonGreen, miRFPnano670, or c-myc is presented in Figure 2d, showing a large gap achieved here between surface-expressing yeasts to those that are not.

Two proteins were excluded from this analysis. The human UNG2 protein showed a substantially larger eUnaG2 signal than c-myc, thus implicating its retention in the secretory pathway. The WD40 domain of ATG16L1 did not express. To uncover the contribution of different reporter brightnesses, we compared FACS fluorescence signals among the eUnaG2 reporter (yellow, pJYDNp), DnbALFA coupled with ALFA-mNeonGreen (green, pJYDN2p), or ALFA-miRFP670nano protein (red), and traditional anti-c-myc (gray, pJYDNp), Figure 2d.

Next, we tested the differences in expression levels between the two basic plasmid arrangements: the protein being N or C terminal to Aga2p. As a fluorescent probe, we used eUnaG2 fused C and N terminal to Aga2p in plasmids pJYDN and pJYDC1, respectively (Figure 2e,f). Large variations in levels of expression were identified among the tested proteins. The dockerin proteins (*Clostridium cellulolyticum*, sequence ID: M93096.1; *Bacteroides cellulosolvens*, AF224509.3; *Clostridium thermocellum*, L06942.1), the receptor IL-20RB, and the angiotensin-converting enzyme ACE2 are preferentially expressed as N-terminal fusions, in contrast to kanamycin resistance protein^48^ and biotin ligase ID2 from *Aquifex aeolicus*(49) which best express as C-terminal fusions. Our inspection of 3D structures for all tested proteins explained only the lack of expression for the kanamycin resistance protein PDB ID 4H05. The strictly conserved C-terminus of this protein was found buried deep inside the structure, not allowing for C-terminus modifications. Therefore, we suggest experimental expression testing for every construct and its expression optimization to achieve good separation between nonexpressing and expressing populations (Figure 2g). Still, if possible, N-terminal expression is preferable as it purges stop-codon insertions in the library without the need for additional steps. The behavior of hard-to-express proteins together with an example of how we overcome this problem is described in detail in the following chapter.

### Part 2: Application of the Enhanced Yeast Display Platform

#### Overcoming the Surface Expression Bottleneck with an Enhanced Yeast Display Platform

One of the largest limitations of yeast display is the requirement for proteins to be correctly processed to the yeast surface. The traditional c-myc or other antibody-based labeling methods do not allow for simple assessment of the protein fraction that is expressed inside the secretory pathway and at the surface. Cell permeabilization and microscopy procedures are needed for such analysis. The alternative dual display, utilizing yeGFP,^50^ shows only the total expression signal, with surface expression deconvolution being possible using antibodies with a different color. The yeast display platform described here enables simple qualitative and quantitative analysis of protein expression on the surface and inside the secretory pathway that can be further coupled with existing tools of computationally assisted design to engineer surface expression of protein targets previously inaccessible in unparalleled time. Following is an example of such a case, the WD40 domain of ATG16L1.^51^

**Figure 3:**
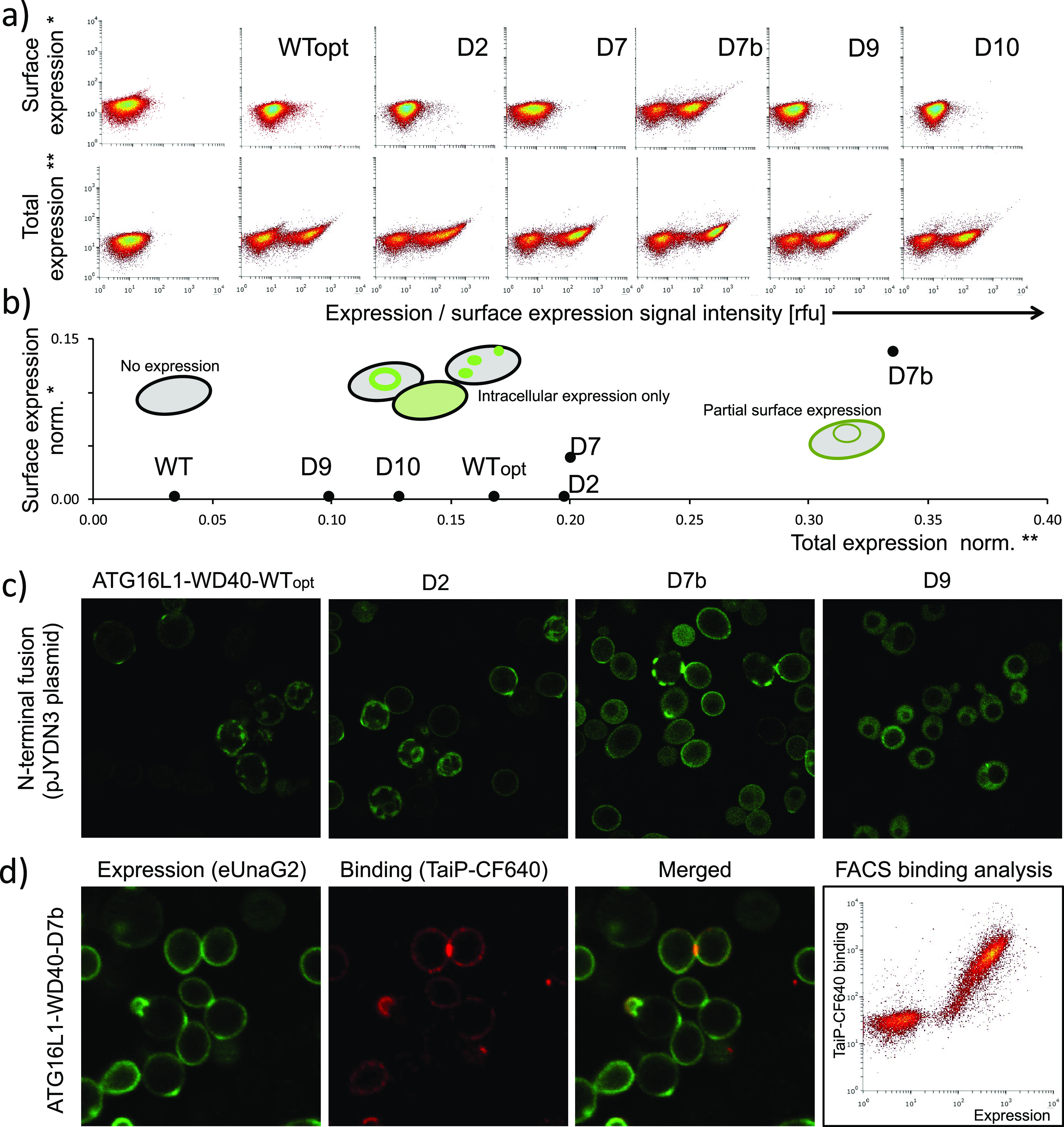
Analysis of surface and total expression as a guide for difficult-to-express proteins. (a) FACS dot plot analysis of surface expression detected with mNeon-DnbALFA (pJYN3 plasmid; upper panels) and the total expression reported by eUnaG2 after incubation with bilirubin. (b) Graph depicting the relation between surface and total expression for different ATG16L1 WD40 domain variants. (c) Fluorescence microscopy analysis of ATG16L1 WD40 domain variant expression. (d) ATG16L1-WD40-D7b variant is binding to its binding partner TaiP protein.

Our initial attempts to express the WD40 domain of ATG16L1 on yeast showed no detectable expression for the wild-type gene. Next, we used a codon-optimized version, showing good expression inside the secretory pathway, but no surface exposure (Figure 3a–c). Following this, we used PROSS stabilization design^39^ to generate protein designs D1–D10 with different amounts of stabilizing mutations (Figure S6). After manual inspection, we decided to test four variants with 10 (D2), 38 (D7), 59 (D9), and 63 (D10) mutations incorporated. Out of the four, only design 7 displayed detectable, albeit weak surface expression (Figure 3a–c). To improve the expression of design 7, we analyzed its cysteines, potential for disulfide bridges, and gain of glycosylations, similarly to the strategy used for eUnaG2 and DnbALFA. Three cysteine residues identified close to the domain surface were mutated to energetically favored residues (Figure S6). The mutagenesis resulted in WD40 domain design 7b that showed 10 times higher surface exposure than the parental design 7. The control binding experiment with ATG16L1 binding partner CT622/TaiP^52^ showed a strong binding signal with 200 nM concentration (Figure 3d) confirming the activity and correct folding of the engineered protein.

#### Selection for Tight Binding Protein-Pairs Using Enhanced Yeast Display

The ultimate aim of yeast display is to generate new activities, such as binding. The here-created pJYDNn and pJYDNg plasmids were used for the generation of six targeted saturation mutagenesis protein libraries (Table 2). The proteins include four previously published scaffold proteins and two new candidates—aminoglycoside-3′-phosphotransferase of type VIII N-terminal domain fragment (Kan-Nfr) and biotin ligase ID2 from *Aquifex aeolicus* C-terminal domain fragment (3EFR-Cfr). Both new candidates were chosen to test the possibility of in vivo enzyme complementation-based experiments that are not covered within the scope of this publication. Among the proteins, Sso7d, Knottin, and GP2 have very high melting temperatures. The other three were prestabilized before library preparation using PROSS calculations and subsequent selection of the PROSS-suggested mutations for the highest level of expression on the yeast surface, using yeast display. This resulted in the incorporation of five stabilizing mutations into s3LYV, seven mutations in 3EFR-Cfr, and 13 mutations in Kan-Nfr (Supporting Information text 4 and Figure S7). The corresponding change in melting temperature upon stabilization was measured using the Prometheus NT.48 for the 3EFR-Cfr and Kan-Nfr-purified wild type and designed proteins. The 3EFR-Cfr and Kan-Nfr-stabilized protein variants were 11 and > 20 °C more stable than the starting proteins. The s3LYV-stabilized protein showed almost 20% higher expression on the yeast surface and better expression in *E.coli* with reduction in inclusion formation.

**Table 2:** pJYDNn Plasmid-Based Yeast Display Libraries

library name	PDB ID	AA	res.	NNK^a^	S^b^	Tm	library size (10^8^)	expr. + cells^e^	mean FL1 [cfu]^f^	ref.
Sso7d	1sso	A1–K62	62	7	N	100	3	17.9%	55,178	(54)
Knottin	1cbh	T1–L36	36	8	N	>80^c^	2	36.8%	124,521	(55)
GP2	2wnm	K35–P79	45	9	N	76^d^	2	32.8%	84,668	(56)
s3LYV	3lyv:A	M14–E60	47	10	Y	ND	5	31.3%	100,966	(56)
3EFR-Cfr	3efr	G188–S233	46	8	11 °C	57	6	32.3%	104,940	This study
Kan-Nfr	4 h05	H(−5)–90P	96	10	>20 °C	86	7	28.6%	48,562	This study

a Number of randomized residues.

b Stabilized protein scaffold: N = no; Y = yes with Tm difference not determined.

c Ref (13).

d Ref (56).

e Percentage of expression positive cells from the total number of single cell events.

f Mean fluorescence of expression positive population.

For library construction, we chose to randomize specific, structurally clustered positions, providing coverage of all possible mutations and combinations rather than random mutagenesis of the complete protein. For Kan-Nfr, positions for library construction were identified by a combination of multiple sequence alignment and *in silico* FoldEX^53^ based saturation mutagenesis (Figure S8). Positions and patches in the structure with large evolutionary variability but low energy variability between mutations were targeted. For 3EFR-Cfr, the small scaffold size made us randomize the β-sheets connecting loops. The six tested scaffolds are comparable in sequence length—all are very small proteins. The outcoming library sizes were comparable, with the number of randomized positions being 7–10 (Table 2). The structure, sequence, and exact position of randomized residues are shown in Supporting information text 4. Library qualities were verified by sequencing 20 randomly selected clones.

#### High-Stringency Selection for Tight Binders

**Figure 4:**
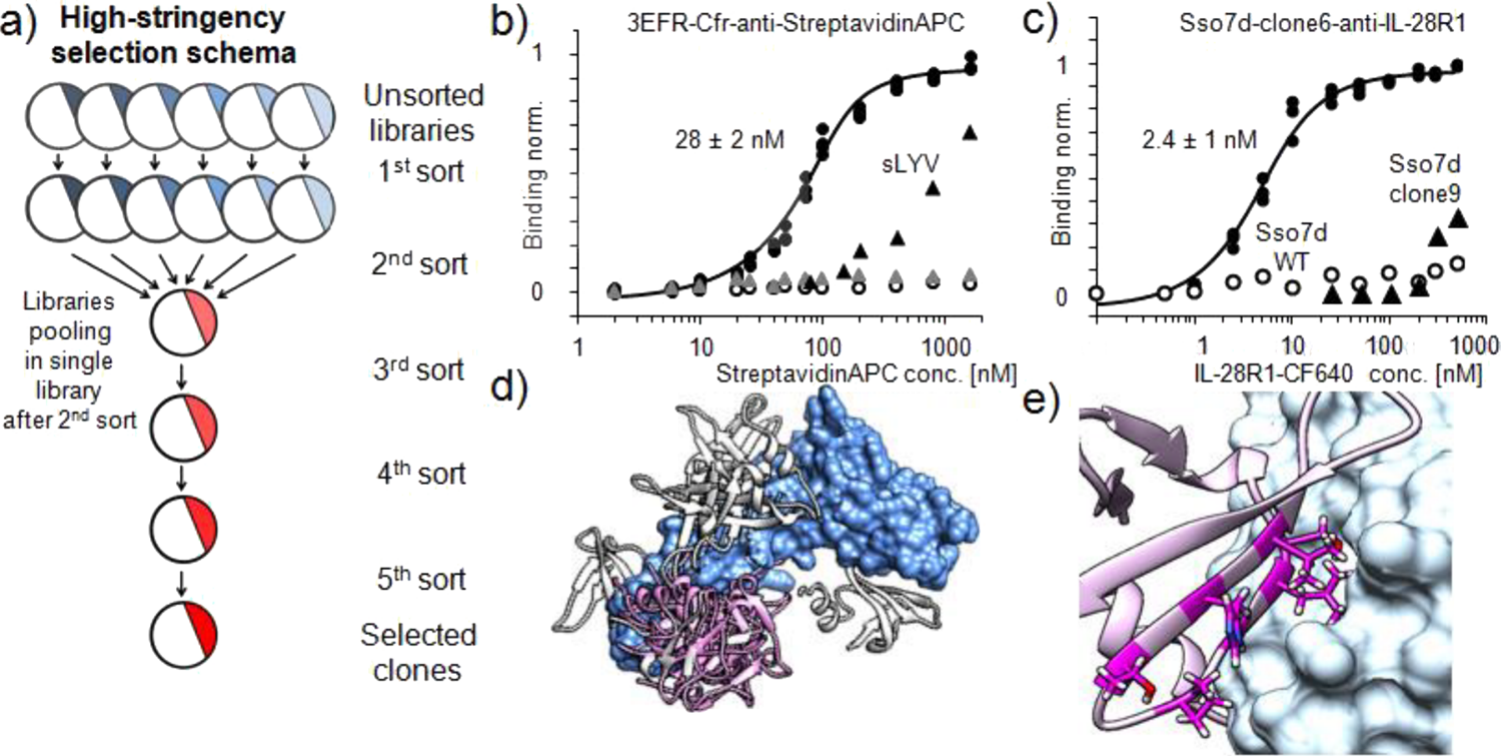
Selection for tight binding by enhanced yeast display. (a) Schema of high-stringency selection used to fish for Streptavidin-APC and IL-28R binding proteins. (b) Binding of Streptavidin-APC selected clones identified after high-stringency selection. High-affinity binder—black circle data points (triplicates); empty circles—wild-type scaffold; black triangles—s3LYV clone; gray triangles—3EFR-Cfr wild type (nonstabilized) with introduced Streptavidin-APC binding residues (single measurement). (c) Binding of IL-28R1 selected clones identified after high-stringency selection. High-affinity binder—black circle data points (clone6, triplicates); empty circles—wild type scaffold; black triangles—Sso7d clone 9; (d) ClusPro docking results depicted on the surface representation of IL-28R (PDB ID: 3og6; in blue). The five best models are highlighted in white and pink for the Sso7d scaffold wild-type and the binding clone6, respectively. (e) Representative model of clone6 binding to IL-28R.

The first selection aimed to find high-affinity binding variants to the commercially available Streptavidin-APC conjugate protein as bait (which naturally does not bind any of these proteins). Our selection strategy was based on preselection against a high concentration of Streptavidin-APC, to decrease the complexity of libraries and subsequent construction of a new pooled library of all preselected scaffold variants (Figure 4a). In the first round, we used all our naïve libraries independently against 1 μM target protein and sorted approximately 1% of cells in the binding/expression quadrant (double-positive cells). In total, we sorted slightly above 10^6^ yeast cells from each library. In the second step, all selected clones from the different libraries were pooled, while keeping the same number of clones from each library (10^7^ per library). Subsequent rounds of sorting were performed with the pooled library against decreasing concentrations of the bait protein—500, 100, 50 nM, and finally 25 nM Streptavidin-APC (Figure 4a). The population of the last sort was plated, and 20 single colonies were screened for binding and sequencing. Among all sequences, we identified one dominant (19/20) 3EFR-Cfr library clone. The equilibrium dissociation constant measured by flow cytometry was calculated to be 28 ± 1.6 nM. The other, single clone was a member of the s3LYV library, and its affinity was estimated to be ≥500 nM (Figure 4b). To exclude the possibility that the 3EFR-Cfr-Anti-Streptavidin targets the APC and not the Streptavidin, we remeasured the binding affinity with the Streptavidin labeled by CF640 dye and obtained similar results to Streptavidin-APC, suggesting no role of APC in binding (Figure 4b). To demonstrate the importance of prestabilization of 3EFR-Cfr prior to selection, we transferred the 3EFR-Cfr-Anti-Streptavidin-APC binding residues to the nonstabilized 3EFR-Cfr and tested its expression and binding properties. The comparison between stabilized and wild-type scaffolds showed complete loss of Streptavidin-APC binding on the wild-type scaffold and a reduction of the clone’s expression by 14% (Figure 4b).

Using the same prey libraries, we repeated the selection against the purified extracellular portion of IL-28R1 (UniProtKB - Q8IU57), the high-affinity receptor for interferon lambda, as bait. In the first round, all libraries were selected independently against 1 μM protein, and then they were pooled and subjected for additional rounds of FACS selection with decreasing concentrations of bait—500, 200, 100, and 50 mM. After the selection, 20 colonies were isolated and screened for binding and sequencing. Two different Sso7d clones were identified. The most prevalent Sso7d clone (no. 6, 19/20 sequences) had a binding affinity of 2.4 ± 1.1 nM as measured by cytometry binding analysis (Figure 4c). The second clone (no. 9) had a *K*_D_ > 1 μM, which corresponds to its incidental presence among sequenced clones.

To uncover the binding interface between our high-affinity binding proteins and their newly evolved targets, we performed protein–protein docking using either the wild-type structures or the corresponding modeled evolved structures (3EFR-Cfr-Anti-Streptavidin-APC, and Sso7d-anti-IL-28R, 20 models each). The difference between the wild-type and mutant docking results was attributed to mutant residues and analyzed manually in greater detail. The analysis resulted in no hits for Streptavidin but a strong signal for the IL-28R binding site which is probably located between D2 domain β-sheets (Figure 4d,e).

## Discussion

Yeast display is the most wildly used in vitro evolution method, with many applications. Here, we aimed at improving the applicability of this method further by revisiting the different steps and optimize them. In the second part of our work, we demonstrate the advantages of the enhanced yeast display platform. First, we shortened the most commonly used pCTcon2 plasmid by 20%, removing unnecessary sequences and replacing the antibiotic resistance to kanamycin. This step increased the efficiency and allowed us to use the restriction-free cloning method^22^ for further steps in the platform development. Next, we changed the labeling procedure of yeast cells for flow cytometry analysis, which currently is laborious with multiple samples and cost-ineffective, requiring multiple washing steps and long incubation times with antibodies.^9^ Previously, GFP^50,57^ and ACP (an orthogonal acyl carrier protein)^4^ were devised for simplification of yeast surface display. The use of GFP in the secretory pathway is connected with multiple impediments such as protein targeting to the vacuole.^58,59^ Some of these difficulties were solved by protein engineering,^25,60−62^ Other problems, like the inability to turn off the fluorescence of GFP, cannot be solved. ACP shows excellent labeling properties, but CoA-biotin and fluorescent CoA-547 and CoA-647 derivatives are needed as well as 1 h of incubation. Overall, none of the alternative techniques became dominant, replacing the method published by Chao et al.^9^

Therefore, we decided to test the performance of several reporter proteins that could improve the yeast display procedure (Figure 1). The differences between the cytoplasmic and surface expressions suggested the protein’s effectiveness in being exposed on the yeast surface. Considering the differences in expression, protein size, and brightness, we identified UnaG, a bilirubin-dependent fluorescent protein, and ALFA-tag binding nanobody as the best candidates for yeast display reporters.

Reporter proteins UnaG and nbALFA were subjected to extensive protein engineering to tailor their properties to fit the yeast surface display platform. Proteins were stabilized using a combination of PROSS calculations,^39^ FACS selections, and N-linked glycosylation site introduction. In total, we introduced 10 mutations in UnaG, with the optimized variant being called eUnaG2 (Figure 1h). From nbALFA, we created the DnbALFA protein, which differs by 10 mutations. The eUnag2 average fluorescence intensity in the expressing population was two and fivefold higher compared to eUnaG^38^ and UnaG, respectively.^26^ The affinity of eUnaG2 for bilirubin was measured and is slightly higher (46 ± 13 pM, Figure S1c) than 98 pM reported for UnaG.^26^ The expression of DnbALFA is almost five times better in mean fluorescence values than the expression of nbALFA on the surface of *S. cerevisiae* EBY100 cells. Interestingly, the protein expression in *E. coli* BL21 (DE3) was also highly enhanced, despite the lack of glycosylation, which resulted in lower melting temperature compared to the wild type. The sodium dodecyl sulfate–polyacrylamide gel electrophoresis (SDS-PAGE) expression analysis showed more than 10-times higher yield for the designed protein variant (Figure S1g). The tailored reporter proteins, eUnaG2 and DnbALFA, were used for the construction of a whole vector platform containing various N and C terminal expression vectors allowing for different expression construct organizations and detection options.

Both reporters enable regulation of their signal and change in the labeling strategy upon need. Bilirubin and the nanobody or its fusion proteins are stable enough to be added directly to the cultivation media. Such addition labels proteins immediately after their maturation and reduces hand-on time dramatically by avoiding the traditional labeling process. A single washing step is usually required. In addition, the two reporters can serve to independently assess the cell surface fraction (DnbALFA) or/and the total protein expression (intracellular and surface). This approach is helpful for rapid in vivo assessment of the secretory pathway retention/surface expression ratio, not easily accessible with previous methods. A comparison between the existing labeling methods and those of the enhanced yeast display platform devised here is shown in Table 3.

**Table 3:** Comparison of Currently Used Yeast Display Methods to the Here-Devised One

	enhanced yeast display platform, this paper	Uchanski et al (2019)^4^	McMahon et al. (2018)^63^	GFP-based methods^50,62^	Boden et al. (1997)^10^
anchor	Aga1p-2p	Aga1p-2p	649AA-tether-GPI	Aga1p-2p	Aga1p-2p
reporter/s (engineered for better surface exp.)	eUnaG2, DnbALFA (yes)	ACP, S6, and SNAP_f_ (no)	no	yeGFP(Yes)	no (tag labeling only)
C terminal expression	three vectors	no	yes	yes, ribosome skipping^58^	1
N terminal expression	three vectors	three vectors	not possible	yes	no
labeling agents (comments)	bilirubin (cheap, easy to obtain), ALFA-tagged-FP (“in lab” preparation)	fluorophore-CoA (limited availability, high price), Sfp synthase (“in lab” preparation)	antibodies	no	antibodies
labeling duration	cocultivation*	> 1 h	> 1.5 h	no	> 1.5 h
label diffusion	prevented by ligand in buffer**	prevented—covalent attachment	yes	no	yes
reporter OFF/ON possibility	yes	yes	yes	no, the GFP signal is always ON	yes
washing steps (expression labeling)	no	yes	yes	no	yes
colors	green / any (“in lab” preparation)	any, fluorophore-CoA dependent	any antibody-conjugate	green only	any antibody-conjugate
assessment of ER retention	yes	no	no	qualitative only by antibodies; not applicable for ribosome skipping	no
comments	The eUnaG2 brightness allows for expression time reduction (∼6 h)			less bright than eUnaG2 (see Figure 1g)	

To demonstrate the performance of our vectors, we initially compared the UNG protein from *E. coli* expression in pCTcon2 and pJYDC labeled by traditional c-myc labeling (Figure 2b). This experiment showed stronger expression in pJYDC, leading to better separation between populations. The signal-to-noise separation was 4.7 times better when we used bilirubin and fluorescence of eUnaG2 than for pCTcon2 and c-myc. Higher separation can be attributed to the strong brightness of eUnaG2 and also to a decrease in background fluorescence. A stronger specific signal translates to a reduced number of consequent selection steps and results in the ability to select for higher binding affinity. Next, we tested different proteins fused to the C-terminus of Aga2p in pCTcon2 and pJYDC (Figure 2c). Protein expression was assessed in parallel by c-myc labeling and by our system reporters eUnaG2 (pJYDC, 12 proteins) or DnbALFA (pJYDC3, five proteins). Because the fluorophores used in our system are brighter, we recorded higher fluorescence intensities compared to c-myc with the eUnaG2 fusion having almost double the fluorescence intensity. To see the impact of fusion location (C or N-terminal), we analyzed the variation in expression of 16 proteins at N and C terminal fusions (pJYDN/pJYDC vector, eUnaG2 reporter). Most of our proteins were expressed in both N and C terminal vectors. Among tested proteins, five proteins were preferentially expressed as N terminal fusions (3 dockerins, IL-20RB, and ACE2 peptidase domain), suggesting that both fusions should be compared.

All proteins chosen in previous analyses were known to be reasonably well expressed. In the next step, we focused on the WD40 domain of the protein ATG16L1 that was not expressed under any conditions tested (Figure 3a, WT). The nucleotide sequence optimized for yeast resulted in the generation of a reasonable signal from the secretory pathway (Figure 3a). With assessing this signal, we designed and tested four variants to improve its solubility. Only one variant showed low surface expression levels (D7) and was further improved by rationalizing its cysteine content. The engineered WD40 domain, designated 7b, showed good expression and binding to its natural partner CT622/TaiP, opening it for further protein engineering (Figure 3). Our platform assessment of the secretory pathway retention/surface expression ratio combined with computer-assisted design showed remarkable strength in protein engineering of hard-to-express proteins reducing the overall hand-on time yet gaining more information. Notably, traditional c-myc labeling would have shown no detectable expression, and the project would be likely terminated.

Overall, all these experiments show that if possible, both intracellular and surface expression inside both N and C terminal plasmids needs to be analyzed for proteins of interest. N-terminal insertion of the library using eUnaG2 as a fluorescent marker for expression is the most desirable method because it purges stop-codons from the library and has the strongest signal. However, if C-terminal fusion is needed, or the use of far red-fluorescence, it is advisable to use the miRFP670nano protein fused to DnbALFA. We took advantage of this system for our work focused on affinity maturation of SARS-CoV2 RBD. Swapping the two reporters among different libraries allowed us to avoid cross-contaminations and simplify the library preparation process by omitting the agarose gel purifications. The presence of template plasmid DNA in the subsequent library was eliminated in the first sort because their expression and binding labeling strategies were incompatible. In addition, the bilirubin added to the bait protein solution prevented diffusion and decrease of the expression signal during washing and bait incubation steps. Together with the much higher affinity of DnbALFA toward ALFA-mNeonGreen, in comparison to c-myc (60 pM versus 10 nM), this allows the use of very low bait concentrations in high volumes, which is required to achieve ultratight binding. This way, we selected a 2.5 pM binder of the SARS-CoV-2 S-protein binding domain toward ACE2 using 500 fM bait protein in 50 mL volumes.^64^

Having created an enhanced yeast display platform, we tested our ability to evolve new protein–protein binding sites. First, we generated six targeted protein libraries using the pJYDNn plasmid for six different scaffold proteins. Among them, four scaffolds were based on literature published data, and two scaffolds were developed by us (Table 2, Figure S7, S8). Stabilization design was implemented for three scaffolds before library construction (s3LYV, 3EFR-Cfr, and Kan-Nfr; Supporting information text 4), which has been shown to have a dramatic impact on proteins’ evolvability^65,66^ by expanding their mutational space. The melting temperature of other scaffolds used in this study was already high (Table 2). The libraries differed in the number of randomized residues and the estimated complexity depending on the design and yeast homologous recombination quality (Table 2). We used stringent conditions and a pooled library after an initial selection round. This experimental strategy allows for different scaffolds to compete with each other in the selection process and selection of the best clones among them. We identified high-affinity binders toward the two baits, streptavidin (28 ± 1.6 nM, 3EFR library clone, Figure 4b) and IL-28R1 (2.4 ± 1.1 nM, Sso7d library clone, Figure 4c). These values are comparable with binders obtained by methods using much higher complexity libraries.^67^ The affinity for IL-28R1 was even higher than the binding affinity with its natural ligands as measured by ELISA assay (15 nM for IFNL1 and 65 nM for IFNL3).^68^ By applying stringent conditions, we identified only a single high-affinity clone for each of the two baits, among the 20 sequenced colonies. This indicates that the best clone over competes others during selection cycles. Both high-affinity binders did not originate from the most complex library. Moreover, the Sso7d library had the lowest proportion of expressing cells, yet gave rise to the best binder to IL-28R1. It demonstrates the importance of parallelization with different libraries to select for high-affinity binders because both chemical and shape are important for binding.^69^ Using *in silico* protein–protein docking, the location of the binding site between Sso7d-anti-IL-28R and IL-28R suggests such complementarity. The Sso7d mutant residues nicely fit in the hydrophobic pocket formed between β-sheets of the IL-28R D2 domain (Figure 4c,d). The fact that we obtained high-affinity binders for both target proteins, done within a week’s time, shows the power of our approach. This would suggest that using the premade libraries of these six small scaffold proteins is sufficient to fish for high-affinity binders for a large variety of proteins. In addition, the results show the importance of using highly stable proteins to increase the success of library selection, as the control experiment where we introduced the wild-type residues back to the Streptavidin-APC binding 3EFR clone showed a complete loss of binding affinity.

## Conclusions

We applied protein engineering and plasmid optimizations to establish an enhanced yeast display method. The simplified selection procedures allow for parallel selection of extremely tight binders, easy labeling strategy alterations to avoid DNA purification steps and prevent cross-contamination and simple assessment of intracellular/surface protein expression ratio. Overall, we constructed 11 different yeast display vectors to enable the N-terminal, C-terminal fusions, and multiple labeling options with two highly engineered reporter proteins—eUnaG2 and DnbALFA tailored to be efficiently processed on the yeast surface. Coupling this platform with automated computational design and mutagenesis represents a new powerful strategy to engineer previously inaccessible proteins, for example, WD40 domain of ATG16L1 within a couple of days. We evaluated the enhanced platform on six different protein libraries and two bait proteins—Streptavidin and IL-28R1. High-affinity binders were selected from a single library which dominated selection after five rounds, suggesting that the high-affinity clone outcompetes the others. The parallelization led to an optimal scaffold selection and isolation of high-affinity binders without the need for high-complexity libraries to be synthesized. In addition to the above-mentioned conclusions, we described two new scaffold proteins for the selection of high-affinity binders that were created by the stabilization of protein fragments and the application of restriction-free cloning for library preparation. Both approaches simplify the process of library design. Our work expands the application range for yeast display and shows that using powerful selection will result in the generation of protein–protein interactions between nonrelated proteins.

## Materials and Methods

### pJYD Yeast Display Backbone Construction and DNA Manipulations

The pJYD vector backbone was assembled by the three-component assembly^22^ from pCTcon2_KAN_ vector.^15^ All components were PCR amplified using KAPA HiFi HotStart ReadyMix (Roche, Switzerland), the template vector was removed by *Dpn*I treatment (NEB, USA) at 37 °C (1–2 h), and subsequently, the amplicons were purified using NucleoSpin Gel and PCR Clean-up kit (Nachery-Nagel, Germany). The assembly reaction was composed of 100 ng of each amplicon and KAPA HiFi HotStart ReadyMix (50 ul reaction mix). The reaction was divided into five aliquots (10 ul) and subjected to assembly PCR (30 cycles; 1 min annealing; 60–70 °C gradient; with 2 °C increments per aliquot; 6 min of polymerization). One μL from PCR reaction aliquots were transformed in electrocompetent *E.coli* Cloni 10G cells (Lucigen, USA). Colonies were screened by colony PCR, and positive colonies were sequenced. The whole plasmid sequence was verified.

Incorporation of further changes into pJYD vectors and other cloning was performed via the restriction-free cloning procedure.^20^ The mutagenic primers were used for amplification of megaprimers. If incorporation or modification of a long sequence was needed, multiple extension PCR amplifications were applied with overlapping primers. All PCR reactions were performed using KAPA HiFi HotStart ReadyMix (Roche, Switzerland). Purified megaprimers (200 ng of DNA, Nachery-Nagel, Germany) were mixed with 20 ng of destination plasmid and subjected to PCR similar to the assembly reaction. The template vector was removed from the PCR mixture by *Dpn*I treatment (NEB, USA) at 37 °C (1–2 h), and 1 μL from PCR reaction aliquots were transformed into electrocompetent *E.coli* Cloni 10G cells (Lucigen, USA). Colonies were screened by colony PCR and sequenced.

### Reporter Genes and Protein Engineering

DNA fragments of mNeonGreen,^24^ yeGFP,^25^ UnaG,^26^ iLOV,^27^ dFP-mini,^28^ GAF-FP,^29^ TDsmURFP^,^^30^ IFP1.4,^32^ miRFP670nano,^33^ nbBC2,^34^ and nbALFA^35^ were ordered from Twist Bioscience (USA) with *S. cerevisiae* codon optimization. Reporter genes were amplified by KAPA HiFi HotStart ReadyMix (Roche, Switzerland) with two sets of primers for intracellular expression (starting with ATG and omitting the Aga2p secretion signal) and yeast surface expression (insertion between *Bam*HI and *Bgl*II sites). PCR products were purified using NucleoSpin Gel and a PCR Clean-up kit (Nachery-Nagel, Germany) and used for amplicon incorporation into destination plasmid by subsequent PCR. The template plasmid molecules were inactivated by *Dpn*I treatment (1 h, NEB, USA) and directly transformed to electrocompetent *E. coli* Cloni 10G cells (Lucigen, USA; 1ul crude reaction mix). Kanamycin-selected clones were screened by colony PCR and verified by sequencing. Correct plasmids were transformed in the EBY100 *S. cerevisiae* strain by the lithium acetate method^70^ and grown on yeast minimal SD-W plates. Reporters’ expressions were analyzed for five colonies using a bdAccuri cytometer (BD Life Sciences, USA).

Protein structures of UnaG,^2^ nbALFA,^35^ and miRFP670nano^33^ were subjected for prediction of stabilizing mutations in Pross server.^39^ Mutagenic primers with suggested mutations were used to generate random libraries via restriction-free method transfer-PCR described by Erijman et al.^41^ Briefly, in the first step, we generate a mix of PCR products by the multiprimer PCR reaction. In the second step, the mix of PCR amplicons was incorporated into pJYD vector by restriction-free cloning PCR, desalted, and electroporated to competent yeast cells. All colonies grown on selection plates were pooled, subjected to mini-prep plasmid purification using a Wizard Plus Minipreps DNA Purification System (Promega, USA), and used as the template in subsequent PCR amplification of the given library. The amplicons were purified (NucleoSpin, Nachery-Nagel, Germany), mixed with the purified cleaved plasmid (pCTcon2KAN vector,^15^*Nhe*I and *Bgl*II), and used for yeast transformation.^7^ Yeast cell cultivation, expression, and selection procedures are described in specific chapters. Cells accompanied by higher fluorescence intensities were sorted and cultivated, and their plasmids were isolated. Isolated plasmids were sequenced, and mutations were analyzed (20 colonies). New genes, including all needed mutations, were purchased from Twist Bioscience (USA), and additional modifications such as gain of N-glycosylation were introduced by site-directed mutagenesis using restriction-free cloning.^22^

### DNA Library Preparation

All libraries were constructed by consecutive extension PCR amplification using KAPA HiFi HotStart ReadyMix (Roche, Switzerland) and NNK-randomized primers (Sigma, USA). The 3EFR-Cfr and GP2 libraries were constructed by extension amplifications of Aga2p gene together with NGL linker. The Knottin library was constructed by the same approach with pJYDNg plasmid. No template genes were used for the construction of these libraries. In contrast, the Kan-Nfr, Sso7d, and s3LYV libraries were amplified from template DNA (pET28bdSUMO plasmids^40^). To reduce the possibility of template amplification, the DNA was gel purified between each PCR extension step. Alterations in codon usage were incorporated into primers to further reduce the template amplification possibility. Library sequences are shown in Supporting information text 4. Purified DNA (10–20 μg per library) was mixed with NdeI and BamHI-cleaved pJYDNn or pJYDNg plasmids (4 μg) and electroporated to EBY100 *S. cerevisiae*.^7^

### Recombinant Protein Expression Systems and Purification

The extracellular part of IL-28R1 (UniProt ID Q8IU57, AA 21–228) was produced by the *Drosophila* S2 expression system. The gene optimized for the *Drosophila* codon usage and extended by C-terminal His-tag was purchased from Life Technologies (DNA String fragments, USA). The DNA fragment was inserted into a pMT-BiP-V5-His_A vector (ThermoFisher) using restriction-free cloning^22^ between the restriction sites *Bgl*II and *Xho*I and verified. Purified plasmid (Plasmid Plus Midi Kit, QIAGEN, Germany) was mixed with selection plasmid pCoBlast (1:10), and the mixture was used for cell transfection by Effectene Transfection Reagent (QIAGEN, Germany) according to the manufacturer’s protocol. A stable cell line was selected using 25 μg/mL Blasticidin S, and protein expression was induced by 1.0 mM CuSO_4_. The protein purification from precipitated cell-culture media was performed on HisTrap HP 5 mL and HiLoad 16/600 Superdex 75 (GE Healthcare, USA) columns by the method described previously.^71^

Protein expressions based on pET26b and pET28bdSUMO^40^ were performed using *E. coli* BL21(DE3) cells and 200 mL 2YT media (1 L Erlenmeyer flasks). Cell cultures were grown (30 °C, 220 rpm) to OD600 = 0.6, then the temperature was lowered to 20 °C, protein expression was induced by 0.5 mM IPTG, and growth continued for 12–16 h. Cells were harvested (6000 g, 10 min), disintegrated by sonication in 50 mM Tris–HCl, 200 mM NaCl buffer (pH 8), and purified by NiNTA agarose. Proteins fused with SUMO (pET28bdSUMO plasmid) were purified by the on-column cleavage method.^47^ Eluted fractions were analyzed on SDS-PAGE gels. For higher purity, HiLoad 26/600 Superdex 75 gel filtration chromatography was applied (PBS buffer).

GST-conjugated TaiP (previously designed as GST-CT622) was produced in *E. coli* and purified as described.^72^

### Yeast Transformation, Cultivation, and Expression Procedures

pJYD plasmids were transformed into *S. cerevisiae* EBY100 by the LiAc–PEG method^70^ and grown on yeast minimal SD-W plates 48–72 h at 30 °C. Liquid SD-CAA cultures (1 mL, composition: 20 g glucose, 6.7 g yeast nitrogen base, 5 g bacto casamino acids, 5.4 g Na_2_HPO_4_, and 8.56 g NaH_2_PO_4_ per 1 L) were inoculated by a single colony and grown overnight at 30 °C (220 rpm). The grown cultures were spun down (3000 g, 3 min), and the culture media were replaced. The expression media, 1/9 media (18 g galactose, 2 g glucose, 8 g yeast nitrogen base, 8 g bacto casamino acids, 5.4 g Na_2_HPO_4_, and 8.56 g NaH_2_PO_4_), were inoculated to OD 1.0 and cultivated at 30 °C overnight (12–14 h, 220 rpm).

### Cocultivation Expression Labeling and Bait Protein Labeling Procedures

According to the detection method, expression media were supplemented either with 1 nM DMSO-solubilized bilirubin (Sigma-Aldrich, USA) or purified 5–10 nM ALFA-tagged fluorescent protein (mNeonGreen) prior to the culture cultivation. The premixed media were not prepared because the stability of bilirubin might be compromised if unprotected from light at room temperature.^73^ After the cultivation, cells were collected (3000 g, 3 min), washed once in ice-cold PBSB buffer, and subjected to analyses. The traditional antibody-based labeling procedure was performed using c-Myc Antibody (9E10, Cat # 626801, BioLegend, USA; incubation 1 h at 4 °C) and Anti-Mouse IgG (Fc specific)-FITC antibody produced in goat (Cat # F4143, Sigma-Aldrich, USA; 30 min at 4 °C).

Bait proteins were labeled by amino-coupling fluorescent dye CF 640R succinimidyl ester (Biotinum, USA) according to the manufacturer’s protocol. Briefly, proteins were transferred to 100 mM bicarbonate buffer (pH 8.2) using Amicon Ultra Centrifugal Filters (3 kDa MWCO, Merck, USA) and mixed with 1: 3 ratio between protein and CF 640R succinimidyl ester dye. The mixture was incubated in the dark at room temperature for 1 h. After the incubation, the solution was transferred in GeBAflex-Midi Dialysis Tubes (8 kDa MWCO, Geba, Israel) and dialyzed twice against 500 mL of PBS buffer at 4 °C (8–12 h). The streptavidin conjugated with APC was purchased commercially (Cat#405207, BioLegend, USA).

### Cytometry Analyses and FACS Sorting

Expressed yeast cells were analyzed using a BD Accuri C6 Plus Flow Cytometer (BD Life Sciences, USA) S3e Cell Sorter device (BioRad, USA). The gating strategy is shown in Figure S9. Green fluorescence channel (FL1-A) was used to record eUnaG2 or FITC signals representing expression positive cells, and a far-red fluorescent channel (FL4-A) recorded CF640R stained proteins binding signals. No compensation was applied. Negative cells, EBY100 cells without plasmid or nonlabeled cells, were used to determine the negative population and set quadrant gating. Quadrants were used to divide the gated cell population into four plots showing negative (LL), nonspecific (UL), expression (LR), and binding (UR) populations.

FACS experiments were performed using a S3e Cell Sorter (BioRad, USA). Cells with surface-expressed proteins, detected *via* eUnaG2, DnbALFA cocultivation labeling, or c-myc antibody labeling, were incubated for 1 h at 4 °C with bait protein and mixed using a lab rotator (5 rpm). Before the sorting, samples were collected by centrifugation (3000 g, 3 min), 1 to 3 times washed with ice-cold PBSB buffer (1 mL), and passed through a cell strainer (40 μM, SPL Life Sciences, Korea).

### Binding Assays and Affinity Curve Determination Using Yeast Display

Aliquots of expressed cells (10^6^) were collected (3000 g, 3 min) and washed in PBSB buffer. The cell pellets were subsequently resuspended in analysis solutions across a range of concentrations. The composition of analysis solutions was as follows: PBSB buffer supplemented with a given concentration of ligand - CF640R labeled bait protein (IL-28R1 or Kan-Nfr) and DMSO-solubilized bilirubin (1 nM final concentration). The aliquots were incubated 1 h at 4 °C and mixed using a lab rotator (5 rpm). Prior to the cytometry analysis, samples were collected by centrifugation (3000 g, 3 min), 1 to 3 times washed with ice-cold PBSB buffer (1 mL), passed through a cell strainer (40 μM, SPL Life Sciences, Korea), and analyzed. The number of washes was increased depending on the background fluorescence. Usually, bait concentrations higher than 100 nM required multiple washing steps. Mean FL4-A values for expressing population subtracted by negative population FL4-A signals were used for determination of binding constant *K*_d_. The fitting of the standard noncooperative Hill equation was performed via nonlinear least-squares regression using Python 3.7. The total concentration of yeast exposed protein was fitted together with two additional parameters describing the given titration curve similarly to.^74^

### Protein–Protein Docking Computations

The structures of PDB ID 1sso (Sso7d) and PDB ID 1swu (Streptavidin-APC) were used for initial structure manipulations before the docking. All nonstandard residues were manually deleted from the PDB files. The mutant residues were introduced (most probable rotamers) and minimized in UCSF Chimera.^75^ The docking models were computed using the ClusPro server.^76^

### Confocal Fluorescence Microscopy

Yeast cells were imaged with an Olympus FluoView FV1000 IX81 Spectral/SIM Scanner confocal laser-scanning microscope (Olympus GmbH, Hamburg, Germany), using 60 X phase-contrast oil-immersion objective, numerical aperture 1.35. The confocal sampling speed was set at 8 μs/pixel. The confocal aperture was fixed at 120 μm for all measurements. The images were collected at 640 × 640 (in pixel) or 52.4 × 52.4 (in μm). dimensions in line sequential mode. Yeast cell samples were put as liquid drops in glass slides with coverslips. To avoid evaporation of the sample, an extra cover glass was placed in the upside direction so that measurements of the samples could be performed in a sandwich mode. Three channels were used for image collections: Fluorescence green channel (excitation at 488 nm and emission at 502–550 nm), fluorescence red channel (detecting the product formation, excitation at 559 nm and emission at 575–675 nm), and a third channel to visualize the transmission image. The laser at 488 nm was operated with 2–5% of its maximum power and the laser at 559 nm was with 20% of its maximum power depending upon the sample expression qualities. For better image quality, 4× to 8× zoom variations have been used.

## Supporting Information Available

pJYD plasmid sequence segment with cytoplasmic and cell surface expression of UnaG wild type, step-by-step protocol, secretory leader and linker optimization, scaffold sequences, randomized positions, library constructs, and stringent selection sequences, eUnaG2 properties and tailoring ALFA-tag binding nanobody for effective yeast display exposure by protein engineering, stability in growth media of the mNeonGreen fluorescence signal over time, secretory leader and linker optimization. pJYDC expression analysis, ALFA-tagged fluorescent proteins and fusions with DnbALFA for yeast display, multiple-sequence alignment of variants of the WD40 domain of ATG16L1, sequence design and testing of the Kan-Nfr scaffold, FoldEX and MSA comparison for mutation site identification in PDB: 4H05, and FACS gating strategy (PDF)
