# Supplementary material for: A Protein-Engineered, Enhanced Yeast Display Platform for Rapid Evolution of Challenging Targets
Source: ACS Synth Biol. 2021 Nov 22;10(12):3445–60. doi: 10.1021/acssynbio.1c00395 (PMC8689690; doi:10.1021/acssynbio.1c00395)
Supplement: Supplementary file 1 — sb1c00395_si_001.pdf [file sb1c00395_si_001.pdf]

Supporting Information to:

A protein engineered, enhanced yeast display platform for rapid evolution of challenging targets

Jiří Zahradník [1], Debabrata Dey [1], Shir Marciano [1], Lucie Kolářová [2], Chloé I. Charendoff [3], Agathe Subtil [3], *Gideon Schreiber [1]

[1] Weizmann Institute of Science, Herzl st. 234, Rehovot 7610001, Israel

[2] Institute of Biotechnology, CAS v.v.i., Prumyslova 595, Vestec 252 50, Prague region, Czech Republic

[3] Institut Pasteur, Unité de Biologie cellulaire de l'infection microbienne, 25 rue du Dr Roux, 75015 Paris, France

(*) Corresponding author: gideon.schreiber@weizmann.ac.il

Content

Supporting information text 1 – 4 and supporting figures 1-8

Supporting information text 1

pJYD plasmid sequence segment with cytoplasmic and cell surface expression of UnaG wild type. Other tested proteins were incorporated at the same position.

```
>UnaG_intracellular_expression
tttcaaaaattcttactttttttttggatggacgcaaagaagtttaataatcatattacatggcattaccaccatata
catatccatatacatatccatatctaatcttacttatatgttgtggaaatgtaaagagccccattatcttagcctaaa
aaaaccttctctttggaactttcagtaatacgcttaactgctcattgctatattgaagtacggattagaagccgccga
gcgggtgacagccctccgaaggaagactctcctccgtgcgtcctcgtcttcaccggtcgcgttcctgaaacgcagatg
tgcctcgcgccgcactgctccgaacaataaagattctacaatactagcttttatggttatgaagaggaaaaattggca
gtaacctggccccacaaaccttcaaatgaacgaatcaaattaacaaccataggatgataatgcgattagttttttagc
cttatttctggggtaattaatcagcgaagcgatgatttttgatctattaacagatatataaatgcaaaaactgcataa
ccactttaactaatactttcaacattttcggtttgtattacttcttattcaaatgtaataaaagtatcaacaaaaaat
tgttaatatacctctatactttaacgtcaaggagaaaaaaccccggatcgaattccctacttcatacattttcaatta
agATGGTCGAAAAGTTTGTTGGCACTTGGAAGATTGCTGATTCTCATAATTTCGGCGAATACTTGAAAGCTATTGGTG
CCCCAAAAGAATTGTCTGATGGTGGTGATGCTACTACTCCAACCTTGTACATTTCTCAAAAGGATGGTGATAAGATGA
CCGTCAAGATTGAAAACGGTCCACCAACTTTTTTGGATACCCAAGTTAAGTTCAAGTTGGGTGAAGAATTCGATGAAT
TCCCATCCGATAGAAGAAAGGGTGTTAAGTCTGTTGTTAACTTGGTCGGTGAAAAATTGGTCTACGTTCAAAAGTGGG
ATGGTAAAGAAACTACTTACGTCAGAGAAATCAAGGATGGCAAGTTGGTTGTTACTTTGACTATGGGTGATGTTGTTG
CCGTTAGATCTTATAGAAGGGCTACTGAAGGATCCGAACAAAAGCTTATTTCTGAAGAGGACTTGTAA
```

- in orange GAL1,10 promoter
- in green UnaG wild type (*S. cerevisiae* optimized)
- in red c-myc sequence

```
>UnaG_surface_expression
tttcaaaaattcttactttttttttggatggacgcaaagaagtttaataatcatattacatggcattaccaccatata
catatccatatacatatccatatctaatcttacttatatgttgtggaaatgtaaagagccccattatcttagcctaaa
aaaaccttctctttggaactttcagtaatacgcttaactgctcattgctatattgaagtacggattagaagccgccga
gcgggtgacagccctccgaaggaagactctcctccgtgcgtcctcgtcttcaccggtcgcgttcctgaaacgcagatg
tgcctcgcgccgcactgctccgaacaataaagattctacaatactagcttttatggttatgaagaggaaaaattggca
gtaacctggccccacaaaccttcaaatgaacgaatcaaattaacaaccataggatgataatgcgattagttttttagc
cttatttctggggtaattaatcagcgaagcgatgatttttgatctattaacagatatataaatgcaaaaactgcataa
ccactttaactaatactttcaacattttcggtttgtattacttcttattcaaatgtaataaaagtatcaacaaaaaat
tgttaatatacctctatactttaacgtcaaggagaaaaaaccccggatcgaattccctacttcatacattttcaatta
agatgcagttacttcgctgtttttcaatattttctgttattgcttcagttttagcacaggaactgacaactatatgcg
agcaaatcccctcaccaactttagaatcgacgccgtactctttgtcaacgactactattttggccaacgggaaggcaa
tgcaaggagtttttgaatattacaaatcagtaacgtttgtcagtaattgcggttctcacccctcaacaactagcaaag
gcagccccataaacacacagtatgtttttaaggacaatagctcgacgattgaaggtagatacccatacgacgttccag
actacgctctgcaggctagtggtggaggaggctctggtggaggcggtagcggaggcggagggtcggctagcCATATGG
TCGAAAAGTTTGTTGGCACTTGGAAGATTGCTGATTCTCATAATTTCGGCGAATACTTGAAAGCTATTGGTGCCCCAA
AAGAATTGTCTGATGGTGGTGATGCTACTACTCCAACCTTGTACATTTCTCAAAAGGATGGTGATAAGATGACCGTCA
AGATTGAAAACGGTCCACCAACTTTTTTGGATACCCAAGTTAAGTTCAAGTTGGGTGAAGAATTCGATGAATTCCCAT
CCGATAGAAGAAAGGGTGTTAAGTCTGTTGTTAACTTGGTCGGTGAAAAATTGGTCTACGTTCAAAAGTGGGATGGTA
AAGAAACTACTTACGTCAGAGAAATCAAGGATGGCAAGTTGGTTGTTACTTTGACTATGGGTGATGTTGTTGCCGTTA
GATCTTATAGAAGGGCTACTGAAGGATCCGAACAAAAGCTTATTTCTGAAGAGGACTTGTAA
```

- in orange GAL1,10 promoter
- Aga2p including its signal peptide is underlined
- in green UnaG wild type (*S. cerevisiae* optimized)
- in red c-myc sequence

Supporting information text 2
Secretory leader and linkers optimization

We initially created a testing vector by introducing UnaG reporter between Aga2p native signal peptide and Aga2p (Supplementary material text 2 Figure S2a). Using this plasmid, we tested the impact of different signal peptides and linkers. We compared three different secretion signals: the natural Aga2p peptide, corresponding to AA 1 – 8, the engineered appS4 [11], and the alpha mating factor 1 leader peptide (MFα1pp). The best performance was observed for the appS4 secretory leader (Figure S2b).

The right peptide linker is important to prevent steric hindrance between the protein of interest and Aga2p and to optimize surface expression [12]. We tested 4 different flexible linkers to secure a distance between yeast agglutinin and the displayed protein (Figure S2c). We tested the commonly used glycine – serine stretch $(GGGGS)_x$ and 3 linkers developed in our lab. Linkers were mined from peptides invisible in protein crystallography structures. It is known that these sequences, not resolved in electron density maps, exhibit substantial flexibility [13]. The best linker was isolated from PDB 3OSS. A free cysteine found in one of our linkers (PDB 5DIS), which showed the lowest level of protein expression, motivated us to mutate this amino-acid residue. Instead of a simple change, we incorporate two serines instead of cysteine and its neighboring amino-acid leucine. This resulted in a gain of the N-glycosylation site in the linker sequence. These alterations resulted in a dramatic increase of eUnaG2 fluorescence (Figure S2c). In parallel, we tested the $(GGGGS)_x$ linker with two asparagines incorporated, which resulted in the highest fluorescence, and was used for all further experiments (designated NGS linker). Overall, the linker engineering showed the importance of N-glycosylation for high yeast surface expression. The final organization used in pJYDN and pJYDC1 plasmids is shown in Figure S2d.

The α-factor leader peptide used in this study slightly differed from the one described in [11].

```
>α factor signal peptide
ATGAGATTTCCTTCAATTTTTACTGCAGTTTTATTCGCAGCATCCTCCGCATTAGCTGCTCCAGT
CAACACTACAACAGAAGATGAAACGGCACAAATTCCGGCTGAAGCTGTCATCGGTTACTCAGATT
TAGAAGGGGATTTCGATGTTGCTGTTTTGCCATTTTCCAACAGCACAAATAACGGGTTATTGTTT
ATAAATACTACTATTGCCAGCATTGCTGCTAAAGAAGAAGGGGTATCTCTCGAGAAAAGAGAGGC
TGAAGCT
```

Supporting information text 3
Step-by-step protocol for enhanced yeast display

Preface: DNA preparation and application of restriction-free cloning for targeted libraries preparation

We took advantage of recent rapid developments in restriction-free cloning methods and applied them to improve and simplify our mutagenic workflow. In the main text we describe the construction of small libraries made of multiple *in silico* predicted mutations. The construction of these libraries was based on the work of Dr. Yoav Peleg [1]. Libraries were created by the multi-primer mutagenic PCR reaction. The principle of this method is based on the generation of different amplicons, so-called mega-primers, and their incorporation in the destination plasmid. A restriction-free approach coupled with error-prone PCR can be adopted in order to mutate selected regions within the gene of interest. The workflow consists of three subsequent PCR steps. In the first step, the random library is generated from the desired segment via error-prone PCR or similar methods. This amplified mutagenized fragment is used as a megaprimer in the next step. The second step is the restriction-free incorporation of library megaprimers in the destination vector, which uses the advantage of the yeast DNA recombination ability and is done in yeasts. In the first step, the reaction template is removed by *Dpn*I cleavage and purification. Next, the whole gene is amplified with recombination overhangs. This approach reduces the necessary steps in traditional methods like overlap-extension PCR, and allow for semi-targeted mutagenesis which is difficult to achieve with different methods.

Note: DNA for library construction needs to be electroporation compatible (eluted with ddH_2O).

Cell maintenance, transformation, expression, and freezing procedures

1) Thaw a frozen aliquot of yeast cells and warm it rapidly up by hand. Remove the cryopreserving media by centrifugation (3000 g, 4 min), re-suspend cells in an appropriate amount of YPD media to create starter culture with OD~0.5 – 1.0, and let them recover overnight in a shaker incubator (30 °C, 220 r.p.m). The viable yeast will grow overnight to an absorbance of approximately 6 (OD600). This initial culture can be stored at 4°C for two weeks without the need for sub-culturing. Selective media (SD media) can be used for *S.cerevisiae* EBY100 cells growth, in place of rich media.

2) For the preparation of yeast electrocompetent or chemically competent cells follow the procedures described by Benatui et al [2] and Gietzet. al [3],[4]. Transformed cells bearing pJYD plasmids should be selected and maintained on tryptophan free media SDCAA/ SD-Trp. The viability and library size can be tested by serial dilutions on SDCAA/SD-Trp plates. Before expression, a liquid starter culture has to be prepared (overnight, 30 °C, 220 r.p.m).

Preparation of electrocompetent cells and electroporation (based on Benatuil et al., 2010 [2], An improved yeast transformation method for the generation of very large human antibody libraries)

A) Grow *S. cerevisiae* EBY100 starter culture overnight to OD600 ~ 3 in YPD media (220 rpm, 30 °C).
B) The next morning inoculate the competent cells culture at an initiate 0.3 OD600 (100 mL of YPD media or depending on the desired library size) and grow them until OD600 = 1.4 – 1.6 (220 rpm, 30°C, timing 4 – 6 h of cultivation).
C) Collect yeast cells (3000 rpm, 3 minutes) and discard the media. Wash the cells twice with 50 ml (1/2 media volume) of ice-cold water and once with an ice-cold electroporation buffer (1 M sorbitol, 1 mM

CaCl2).Note: We recommend high quality grade water (such as HPLC grade) for washing and buffer preparation to improve the transformation efficiency.
D) Resuspend the yeast cells pellet in 25 mL (1/4 media volume) of 0.1 M LiAc, 10 mM DTT and condition them in shaker incubator (220 rpm) for 30 minutes at 30 °C.
E) Collect conditioned cells by centrifugation (3000 rpm, 3 minutes), wash once with 50 ml ice-cold electroporation buffer, and re-suspended the cell pellet with the electroporation buffer to the final volume (1.2 – 2 ml is optimal for yeast cells grown in 100 mL of YPD which corresponds to approximately 1.6×10^9 cells/ml). Now cells are ready for electroporation.
F) Add DNA to your yeast sample, single electroporation in 2 mm electroporation cuvettes requires 400 μl of yeast cells suspension. Electroporate cells at 2.5 kV and 25 μF. Typical time constant ranges from 3.0 to 4.5 milliseconds. (Note: Dilution with the electroporation buffer can be used to reduce conductivity.
G) Recover cells by incubation in 10 ml of 1:1 mix of 1 M sorbitol at shaker incubator (30°C, 220 rpm) for 1 – 2 h.
H) Collect cells by centrifugation (3000 rpm, 3 minutes), precisely remove the supernatant, resuspend cells in the appropriate amount of selective media SDCAA (the same volume as initial YPD culture) and grow them 16 – 24 h.

3) Start the expression culture by pelleting 1 ml of starter culture cells (3000 g, 4 min). Remove the media and resuspend cells in 1/9 expression media to OD = 1. Cell expression conditions and temperatures may vary depending on protein and selection purposes (24 – 72 h, 20 – 37°C). Generally, proteins that are hard to express require lower starting OD (0.5 – 0.8), lower expression temperatures, and longer incubation times. The exact values have to be experimentally determined. Do not allow cells to reach the late stationary phase (OD >8) at which the surface expression drops and the protein quality may decrease due to media acidification. Cell co-cultivation labelling allowed in enhanced yeast display is described in detail in the chapter dedicated to labelling.
4) For *S. cerevisiae* EBY100 cells, electrocompetent cells, transformed cells and libraries freezing procedures follow the procedure described by Suga et al. [5] with 2 M sorbitol which we discover to be more efficient than traditional procedures based on glycerol or DMSO.

Co-cultivation labelling of yeast cells for enhanced yeast display
The labelling procedures of enhanced yeast display are versatile and depending on the plasmid used in the experiment. Here we will introduce the most common procedures utilizing the eUnaG2, DnbALFA and ALFA tag binding fusion proteins. Notes related to specific plasmids are listed at the end of this part of protocol.

5) eUnaG2 reporter co-cultivation labelling procedures (plasmids pJYDN, pJYDNp, pJYDNg, pJYDNgp, pJYDN3 and pJYDN3p). Prepare the free-bilirubin (Sigma, SKU 14370) in DMSO solution (2 μM). This solution should be kept at -20 °C and can be used for a period of at least 6 months. Prolonged incubation at room temperature or in light will greatly reduce its stability. The solution is 2000-times concentrated stock and can be directly added to the inoculated expression culture before cultivation. We do not recommend the preparation of 1/9 media with bilirubin in advance since prolonged storage at 4 ^{0}C may compromises its stability and decrease the result reproducibility. Expression culture without bilirubin can be used for alternative labelling purposes e.g. intracellular/surface expression analysis. The absence of bilirubin in cultivation media can be replaced by its addition at least an hour before the harvesting or by direct 15 min labelling on ice with PBSB supplemented with 10 nM bilirubin. Bilirubin itself is not fluorescent

in DMSO solution. A slight change of media color is neither compromising the fluorescence analysis nor viability of cells.

6) DnbALFA reporter nanobody co-cultivation labelling procedures (corresponds to plasmids pJYDN2 and pJYDN2p). The DnbALFA reporter enables any color labelling with either purified ALFA-tagged fluorescent proteins or fluorescently labelled peptides. The co-cultivation labelling has to be optimized for the labelling agent used (stability). The optimal concentration for purified ALFA-tagged mNeonGreen was 5 nM. The co-cultivation labelling can be replaced by more traditional procedures with the addition of a labelling agent at least an hour before the harvesting or by 15 min labelling on ice.

Note 1 – C-terminal plasmids pJYDC1-C3: Although the introduction of HDEL endoplasmic retention - sequence largely reduces the false positive signal, it is still leaky and expression can be recorded from the empty plasmid. Therefore the presence of protein of interest on the yeast surface needs to be carefully verified. When using a binding partner and selection for tighter binding, than the binding signal itself is a good confirmation. The more complicated situation is when selecting for non-existing interaction or expression only. Under such condition we recommend tag swopping procedure between an empty plasmid and correctly assembled plasmid. The identification of tag on the surface of yeast which is different from the original plasmid is a good confirmation. Still, if possible, N-terminal plasmid is a superior alternative.

Note 2 – protein retention analysis: The plasmid pJYDN3 is dedicated specifically for protein retention analysis: Two cultures should be expressed simultaneously – one with the addition of bilirubin (total eUnaG2 signal) and the second one bilirubin-free. The bilirubin-free culture surface expression is subsequently visualized by cell labelling procedure with PBSB supplemented with purified 5 mM eUnaG2-DnbALFA (cell-surface signal only, 30 min on ice, two washes with PBSB buffer). The same fluorescence signal reporter ensures easy extracellular/intracellular signal deconvolution without the need for fluorescence intensity calibration required with different fluorescent probes e.g. using antibody-based c-myc labelling.

Note 3 – parallelization with different labelling: We recommend using of different labelling strategies with different libraries run in parallel to prevent cross-contaminations. Similar approach can be adopted for the consecutive libraries as was shown previously [6].

Sorting process

7) The expression labelling strategy and the target labelling have to be compatible. We recommend using amino-reactive succinimidyl ester-based dye labelling instead of biotinylation and coupling with streptavidinAPC, especially for small target proteins to prevent false-positive (streptavidinAPC binding) or false-negative results (sterical hindrance).

Grow an expression culture of yeast with the population size larger than 10-times the library complexity. Generally, for the library with 10^8 variants 1 ml of starter culture (OD 3 - 5) covers the complexity with large excess.

Re-suspend cells in an appropriate volume of PBSB buffer supplemented with the labelled bait protein. The volume and incubation time is dependent on the affinity [7] or selection strategy [8]. Larger volumes (> 1 ml) are needed to avoid bait protein depletion with concentrations lower than 1 - 2 nM (To test this effect, incubate expressing yeasts with 2 different volumes of bait protein and compare the recorded fluorescence.). Incubate labelling mixture on rotator shaker to ensure homogenous binding conditions (5 – 30 rpm, 4 – 20°C). After bait protein incubation

wash cells once with PBSB buffer (3000 g, 4 min). Keep cells pelleted prior to sorting to prevent dissociation.

8) Use a small sample of your cells to adjust your FACS sorter device setting, gating and/or compensation strategies for your experiment. If the negative population (not expressing population) shows higher signals than expected, which is a sign of nonspecific binding, wash your cells again with PBSB buffer (3000 g, 4 min). Choose the sorting gate in accordance with your selection approach – usually the double-positive quadrant. Different strategies were shown in Zahradnik et al. 2021 [6]. The general rule for sorting strategy is to start with a broad gating strategy (select 5 – 10 % of the population) and increase stringency in subsequent rounds up to the top 0.1 % of the double-positive population. This strategy prevents the loss of unique clones that did not express their properties due to competition with a large excess of other yeasts. We recommend sorting a minimum of 20k cells. After sorting, concentrate cells to remove the sheath fluid by centrifugation (3000 g, 4 min) and resuspend cells in an appropriate amount of SDCAA media (1 ml is sufficient) and grow them for 24 – 48 h at 30 °C (220 r.p.m). Note: Less than 200 000 yeast cells are not forming clearly distinguishable pellet. Last 50 µl can be left in the tube to prevent cell loss.

Repeat growth/sorting cycles until the population is substantially enriched.

Single-cell isolate characterization

9) After the population enrichment for the desired cells (three to five rounds of FACS) the verification of multiple single clones usually takes place before more in-depth analysis. This step comprises of flow cytometry analysis that confirms binding to the selected target and sequencing of selected clones. The sequencing steps can be significantly simplified over the traditional methods which require enzymatic lysis, commercial yeast plasmid purification kits, *E.coli* transformation, and additional mini-prep purification [7].

Two simple methods are used frequently in our lab. The slower method with higher yields utilizes enzymatic lysis with lyticase from *Arthrobacter luteus* (commercially available). The cell pellet (0.5 ml of grown culture) is resuspended in 50 µl of lyticase solution (2 kU/ml, PBS + 50 % glycerol) and incubated 2 h to overnight at room temperature. The suspension is then subjected to standard mini-prep kit isolation procedure.

The second method is very fast with lower yields. We have discovered that the method for yeast chromosomal DNA isolation published by Lõoke et al. [9] can be modified for yeast plasmid preparations. The procedure consists of lithium acetate SDS cell lysis and ethanol precipitation. The resulting pellet contains total DNA (genomic and plasmid) from the sample and can be further subjected to a standard mini-prep isolation procedure. The obtained DNA is sufficiently pure to be directly used in PCR applications, for transformations, or as a starting material for mini-prep procedures.

The process of clone sequencing can be further accelerated by direct sequencing of colony PCR product, treated with ExoSAP enzyme mix [10].

Sequencing primers for pJYDN plasmid	
Forward (GaL1b)	CCTCTATACTTTAACGTCAAGGAG
Reverse (seq_R1)	CGGTGAAAATAGATGGGAACCTC
Sequencing primers for pJYDC plasmids	
Forward (C_seq_F)	GCAGCCCCATAAACACACAGTATG
Reverse (pCT_seq_R)	CATGGGAAAACATGTTGTTTACGGAG

Media compositions

YPD media (1 L)

10 g yeast nitrogen base
20 g peptone
20 g D-(+)-glucose

SDCAA (1 L)

20.0 g glucose
6.7 g yeast nitrogen base
5.0 g bacto-casamino acids
5.4 g Na_2HPO_4
8.56 g NaH_2PO_4

1/9 expression media(1 L)

18.0 g galactose
2.0 g glucose
8.0 g yeast nitrogen base
8.0 g bacto-casamino acids
5.4 g Na_2HPO_4
8.56 g NaH_2PO_4

Amino acid composition of SD-W (1 L)

20 mg Adenine
20 mg Arginine
80 mg Aspartic acid
20 mg Histidine
30 mg Isoleucine
100 mg Leucine
30 mg Lysine
20 mg Methionine
50 Phenylalanine
200 mg Threonine
20 mg Tryptophane

30 mg Tyrosine
20 mg Uracil
150 mg Valine
6.7 g yeast nitrogen base

Protocol-at-a-glance for one standard yeast display cycle

Day 1
- EBY100 starter (10 ml YPD, grow overnight)
- DNA preparation (PCR amplification of library, plasmid cleavage with restriction endonucleases - recommended *Nde*I and *Bam*HI)

Day 2
- DNA preparation (PCR product and plasmid clean-up, elute DNA with ddH_2O)
- Electrocompetent cells preparation (100 ml YPD)
- Library construction *via* electroporation (prepared clean DNA, 100 ml SDCAA)

Day 3
- Expression culture (spin 1 ml of library (3000 g, 3 min), resuspend in 5 ml of 1/9 media, cultivate 24 – 48 h at 20°C)
- Co-cultivation labelling (add bilirubin or ALFA-tagged proteins to you culture according to your strategy)

Day 4
- Incubation with bait protein (spin cell aliquot (1 ml) and resuspend in PBSB buffer with bait protein (volume and incubation time depends on affinity) and optionally with expression labelling compound to prevent dissociation).
- Wash cells once with PBSB before sorting to reduce the background
- Sort cells according to your strategy (20k recommended minimum)
- Expand sorted cells (1 – 5 ml of SDCAA media, 24 h at 30°C)

Supporting information text 4

Scaffold sequences, randomized positions, library constructs, and stringent selection sequences

All scaffolds were optimized for expression in *S. cerevisiae*. The first nucleotide sequences were used for scaffold expression verification and also for control expression in *E.coli*. The amino-acids highlighted by grey background were randomized. Libraries sequences are including overhangs for homologous recombination (underlined). Some of them differ in non-randomized nucleotides compared to nucleotide sequences only. This difference is only on the codon usage level and was incorporated during library construction via PCR reactions. The arginine highlighted by both grey background and bold letters incorporated in 3EFR-Cfr-Anti-Streptavidin did not originate in the randomized residues.

List of stabilizing mutations in 3EFR-Cfr compared to wild-type (PDB: 3efr structure numbering):
E190R, L194I, K198E, K202I, K209N (randomized position), L215E, E217D;

```
> 3EFR-wild type
GEEVKLLGEGKITGKLVGLSEKGGALILTEEGIKEILSGEFSLRRS
> 3EFR-Cfr scaffold amino-acid sequence
GERVKLIGEGEITGILVGLSENGGALIETDEGIKEILSGEFSLRRS
```

>3EFR-Cfr scaffold nucleotide seq
GGTGAACGCGTTAAACTGATCGGTGAAGGTGAAATTACAGGTATTCTGGTTGGTCTGAGCGAAAA
TGGTGGTGCACTGATTGAAACCGATGAAGGCATTAAAGAAATTCTGAGTGGTGAATTTAGCCTGC
GTCGTAGC

> 3EFR-Cfr scaffold amino-acid sequence
GERVKLIGEGEITGILVGLSENGGALIETDEGIKEILSGEFSLRRS

>pJYDNn-3EFR-Cfr Library
GCTGCTTCTTCTGCTTTGGCTGCTCCAGCTAATGGTGGTGAACGCGTTAAACTGATCGGTNNKGG
TGAAATTACAGGTATTCTGGTTGGTCTGAGCNNKNNKNNKGGTGCACTGATTGAAACCGATGAAG
GCATTAAAGAAATTNNKNNKNNKNNKTTTAGCCTGCGTCGTAGCTCTGGTGGAGGTGGTTCGGGC
GGAGGTGGTAACGGATCTAATGG

- template pJYDNn plasmid
3EFR_lib_F1:GGTGCACTGATTGAAACCGATGAAGGCATTAAAGAAATTNNKNNKNNKNNKTT
TAGCCTGCGTCGTAGCGGATCCGGATCCGTTTCTGCACAGGAACTGACAACTATATGCG
3EFR_lib_F2:GGTGAACGCGTTAAACTGATCGGTNNKGGTGAAATTACAGGTATTCTGGTTGG
TCTGAGCNNKNNKNNKGGTGCACTGATTGAAACCGATGAAGGC

3EFR_lib_F3:GCTGCTTCTTCTGCTTTGGCTGCTCCAGCTAATGGTGGTGAACGCGTTAAACT
GATCGGTG
Aga2p_R: GTGTTTATGGGGCTGCCTTTGC

List of stabilizing mutations in Kan-Nfr compared to wild-type (PDB: 4H05 structure numbering):
G10R (randomized position), C15Y, V18E, V19P, G36N, G49A, V50A, V60E, E64R, V65Q, G75Y, E79D, R80G;

The comparison of 4H05 wild-type and scaffold sequence is shown in Figure S4a

> Kan-Nfr scaffold nucleotide seq
ATGCATCATCATCACCATCACATGGATGATGCACTGCGTGCCCTGCGTCGTCGTTATCCGGGTTA
TGAATGGGAACCTGTTGAAGATGGTGCAAGCGGTGCCGGTGTTTATCGTCTGCGTGGTGGTAATC
GTGAACTGTTTGTTAAAGTTGCAGCACTGGGTGCAGCAGCAGGTCTGCTGGGTGAAGCAGAACGT
CTGGAATGGCTGGCACGTCAGGGTATTCCGGTTCCGCGTGTTGTGGAATATGGTGGTGATGACGG
TGTTGCATGGCTGGTTACCGAAGCA

> Kan-Nfr scaffold amino-acid sequence
MHHHHHHMDDALRALRRRYPGYEWEPVEDGASGAGVYRLRGGNRELFVKVAALGAAAGLLGEAER
LEWLARQGIPVPRVVEYGGDDGVAWLVTEA

>pJYDNn Kan-Nfr scaffold library
GCTGCTTCTTCTGCTTTGGCTGCTCCAGCTAATGGTCATATGCACCACCATCATCACCATATGNN
KNNSGCACTGNNKNNSCTGNNKNNSNNKTATNNSGGTTATNNKNNSGAACCTGTTGAAGATGGTG
CAAGCGGTGCCGGTGTTTATCGTCTGCGTGGTGGTAATCGTGAACTGTTTGTTAAAGTTGCAGCA
CTGGGTGCAGCAGCAGGTCTGCTGGGTGAAGCAGAACGTCTGGAATGGCTGGCACGTCAGGGTAT
TCCGGTTCCGCGTGTTGTGGAATATGGTGGTGATGACGGTGTTGCATGGCTGGTTACCGAAGCAT
CTGGTGGAGGTGGTTCGGGCGGAGGTGGTAACGGATCTAATGG

Kan-Nfr _F1:
GTCATATGCACCACCATCATCACCATATGNNKNNSGCACTGNNKNNSCTGNNKNNSNNKTATNNS
GGTTATNNKNNSGAACCTGTTGAAGATGGTGCAAGCG
Kan-Nfr _F2:
GCTGCTTCTTCTGCTTTGGCTGCTCCAGCTAATGGTCATATGCACCACCATCATCACCATATG
Kan-Nfr _R:
CGCATATAGTTGTCAGTTCCTGTGCAGAAACGGATCCTGCTTCGGTAACCAGCCATGC

>Knottin scaffold nucleotide seq
ACCCAATCTCATTATGGTCAATGTGGTGGTATTGGTTATTCTGGTCCAACTGTTTGTGCTTCTGG
TACTACTTGTCAAGTTTTGAACCCTTACTACTCCCAATGCTTG

> Knottin scaffold amino-acid sequence
TQSHYGQCGGIGYSGPTVCASGTTCQVLNPYYSQCL

> pJYDNg Knottin scaffold library
GCTGCTTCTTCTGCTTTGGCTGCTCCAGCTAATGGTCATATGACCCAATCTCATTATGGTCAATG
TGGTGGTNNKNNKNNKNNKNNKCCAACTGTTTGTGCTTCTGGTACTACTTGTCAAGTTTTGNNKN
NKNNKTACTCCCAATGCTTGTCCCAAAAGTTGGACATCAACTTG

- template pJYDNg plasmid
Knott_F1:GTGCTTCTGGTACTACTTGTCAAGTTTTGNNKNNKNNKTACTCCCAATGCTTGTCC CAAAAGTTGGACATCAACTTG
Knott_F2:GACCCAATCTCATTATGGTCAATGTGGTGGTNNKNNKNNKNNKNNKCCAACTGTTT GTGCTTCTGGTACTACTTGTCAAG
Knott_F3:
GCTGCTTCTTCTGCTTTGGCTGCTCCAGCTAATGGTCATATGACCCAATCTCATTATGGTCAATG TG

>GP2 scaffold nucleotide seq
AAATTTTGGGCCACTGTTGAATCCTCTGAGCATTCCTTTGAAGTTCCGATATACGCGGAGACCCT AGACGAAGCACTAGAATTGGCCGAATGGCAATATGTACCGGCTGGATTTGAGGTGACGAGGGTGA GGCCC

>GP2 scaffold amino-acid sequence
KFWATVESSEHSFEVPIYAETLDEALELAEWQYVPAGFEVTRVRP

> pJYDNn GP2 scaffold library
<u>GCTGCTTCTTCTGCTTTGGCTGCTCCAGCTAATGGTCATATG</u>AAATTTTGGGCCACTGTTGAANN KNNKNNKNNKTCCTTTGAAGTTCCGATATACGCGGAGACCCTAGACGAAGCACTAGAATTGGCCG AATGGCAATACNNKNNKNNKNNKNNKGAGGTGACGAGGGTGAGGCCC<u>GGATCCTCTGGTGGAGGT GGTTC</u>

- template pJYDNn plasmid
GP2_F1:CTAGAATTGGCCGAATGGCAATACNNKNNKNNKNNKNNKGAGGTGACGAGGGTGAGGC CCGGATCCTCTGGTGGAGGTGG
GP2_F2:
CTTTGAAGTTCCGATATACGCGGAGACCCTAGACGAAGCACTAGAATTGGCCGAATGGCAATAC
GP2_F3:
GTCATATGAAATTTTGGGCCACTGTTGAANNKNNKNNKNNKTCCTTTGAAGTTCCGATATACGCG G
GP2_F4:
GCTGCTTCTTCTGCTTTGGCTGCTCCAGCTAATGGTCATATGAAATTTTGGGCCACTGTTG

List of stabilizing mutations in s3LYV compared to wild-type (PDB: 3lyv:A structure numbering):
V16S, I32V, D38T, A40Q, T41V;

> 3LYV-wild type
MDVEEARLQMELLGHDFFIYTDSEDGATNILYRREDGNLGLIEAKLE
> s3LYV scaffold amino-acid sequence
MDSEEARLQMEL<u>LGHDF</u>FVYTDSETGQVNILY<u>RREDG</u>NLGLIEAKLE

>s3LYV scaffold nucleotide seq
ATGGATTCTGAAGAGGCTAGATTGCAGATGGAATTATTGGGTCATGATTTCTTCGTTTACACCGA TTCTGAAACCGGTCAAGTTAATATCTTGTACAGAAGAGAGGACGGTAACTTGGGTTTGATTGAAG CTAAATTGGAA

>s3LYV scaffold amino-acid sequence
MDSEEARLQMELLGHDFFVYTDSETGQVNILYRREDGNLGLIEAKLE

> pJYDNn s3LYV scaffold library
GCTGCTTCTTCTGCTTTGGCTGCTCCAGCTAATGGTCATATGGATTCTGAAGAGGCTAGATTGCA
GATGGAATTANNKNNKNNKNNKNNKTTCGTTTACACCGATTCTGAAACCGGTCAAGTTAATATCT
TGTACNNKNNKNNKNNKNNKAACCTGGGTTTGATCGAGGCTAAATTGGAAGGATCCTCTGGTGGA
GGTGGTTC

LYV_F1:AACCTGGGTTTGATCGAGGCTAAATTGGAAGGATCCTCTGGTGGAGGTGGTTC
LYV_F2:CGTTTACACCGATTCTGAAACCGGTCAAGTTAATATCTTGTACNNKNNKNNKNNKNNK
AACCTGGGTTTGATCGAGGC
LYV_F3:CATATGGATTCTGAAGAGGCTAGATTGCAGATGGAATTANNKNNKNNKNNKNNKTTCG
TTTACACCGATTCTGAAACCG
LYV_F4:
GCTGCTTCTTCTGCTTTGGCTGCTCCAGCTAATGGTCATATGGATTCTGAAGAGGCTAGATTGC

> sso7d scaffold nucleotide seq
GCTACCGTTAAGTTTAAGTACAAGGGTGAAGAAAAGCAGGTCGACATTTCCAAGATTAAGAAGGT
TTGGAGAGTCGGCAAGATGATTTCTTTCACTTATGACTTAGGTGGTGGTAAGACTGGTAGAGGTG
CTGTTTCTGAAAAAGATGCCCCAAAAGAACTGTTGCAAATGTTGGCTAAACAGAAGAAG

> sso7d scaffold amino-acid sequence
ATVKFKYKGEEKQVDISKIKKVWRVGKMISFTYDLGGGKTGRGAVSEKDAPKELLQMLAKQKK

> pJYDNn sso7d scaffold library
GCTGCTTCTTCTGCTTTGGCTGCTCCAGCTAATGGTCATATGGCTACCGTTAAGTTTAAGTACAA
GGGTGAAGAAAAGCAGGTCGACATTTCCAAGATTNNKNNKGTTNNKAGANNKGGCAAGNNKATTN
NKTTCNNKTATGACTTAGGTGGTGGTAAGACTGGTAGAGGTGCTGTTTCTGAAAAAGATGCCCCA
AAAGAACTGTTGCAAATGTTGGCTAAACAGAAGAAGGGATCCTCTGGTGGAGGTGGTTCGGGCGG
AG

JZ_sso7d_F:
CTTTGGCTGCTCCAGCTAATGGTCATATGGCTACCGTTAAGTTTAAGTACAAGGGTG
JZ_sso7d_lib1_F1:GTTTAAGTACAAGGGTGAAGAAAAGCAGGTCGACATTTCCAAGATTNN
KNNKGTTNNKAGANNKGGCAAGNNKATTNNKTTCNNKTATGACTTAGGTGGTGGTAAGACTGG
JZ_sso7d_lib1_F2:GCTGCTTCTTCTGCTTTGGCTGCTCCAGCTAATGGTCATATGGCTACC
GTTAAGTTTAAGTACAAGGGTGAAGAAAAGC
JZ_sso7d_R:
CTCCGCCCGAACCACCTCCACCAGAGGATCCCTTCTTCTGTTTAGCCAACATTTGCAAC

Stringent selection

>3EFR-Cfr-Anti-Streptavidin
GERVKLIGEG**R**ITGILVGLSGQFGALIETDEGIKEIWVPGFSLRRS

>sso7d-Anti-IL-28R1_clone_6
ATVKFKYKGEEKQVDISKISLVGRPGKTIHFVYDLGGGKTGRGAVSEKDAPKELLQMLAKQKK

>sso7d-Anti-IL-28R1_clone_9
ATVKFKYKGEEKQVDISKIQVVARWGKRIAFGYDLGGGKTGRGAVSEKDAPKELLQMLAKQKK

Supplementary material Figures

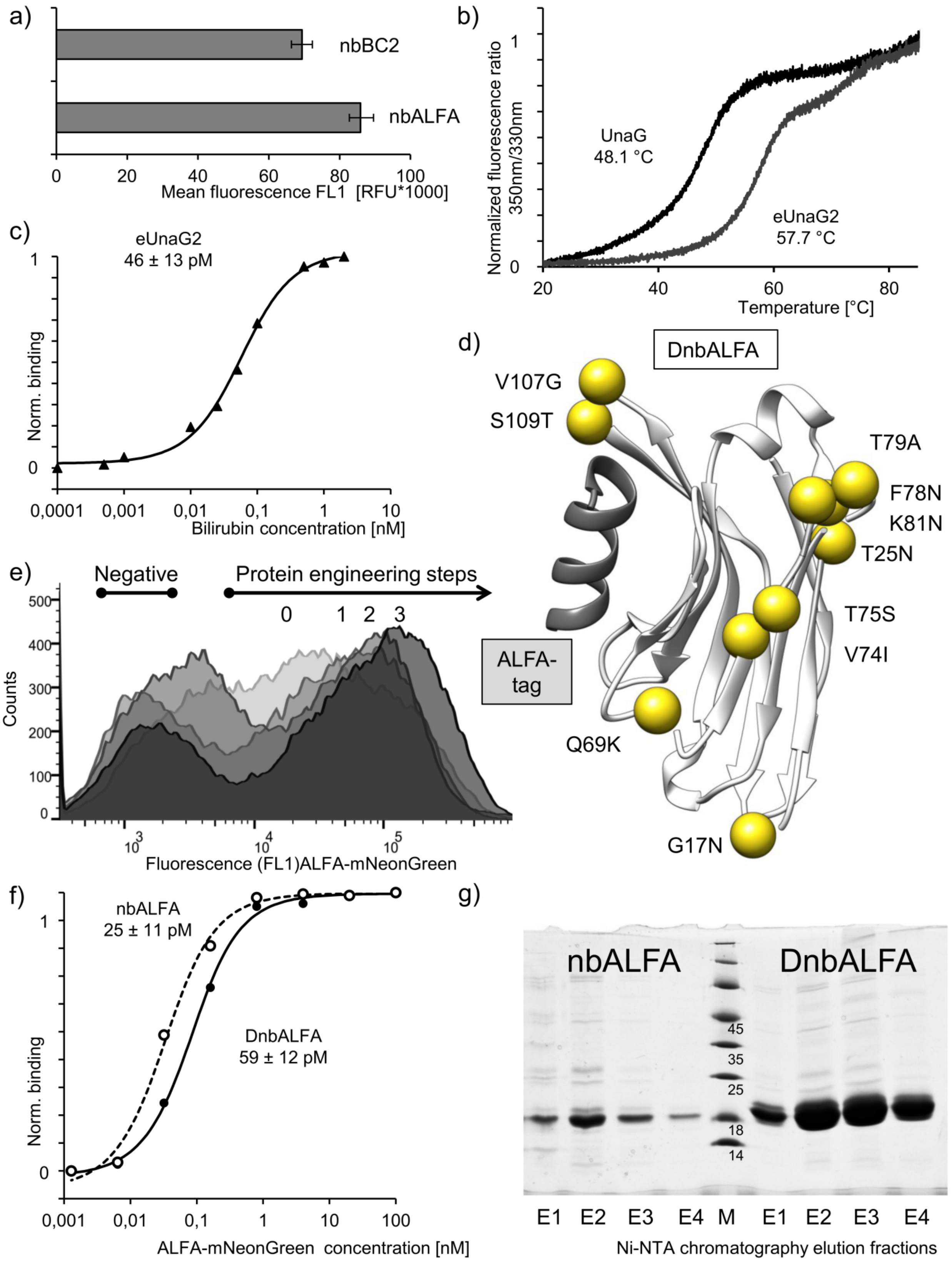

Figure S1 – eUnaG2 properties and tailoring ALFA-tag binding nanobody for effective yeast display exposure by protein engineering. a) Bilirubin titration curve of eUnaG2. The affinity is higher than the affinity reported for UnaG (98 pM) [14] b) Melting of UnaG and eUnaG2 measured by using DSFnano Prometheus NT.48 instrument (Nanotemper Technologies). c) Mutations in designed DnbALFA introduced by protein engineering compared to original nbALFA (PDB id: 6I2G). d) Comparison of surface expression of tested nanobodies fused to the C-terminus of Aga2p measured by traditional c-myc labeling between nbBC2 and nbALFA. e) Flow cytometry histograms showing the green fluorescence signal (FL1 channel) of different variants of nbALFA exposed on yeast surface and visualized by using Anti-c-myc antibody labelling. 0 – wild-type, 1 – Q69K, 2 – combining all predicted stabilization mutations, 3 – combining two N-glycosylation gaining mutations (G17N, T25N) with all stabilizing mutations; e) Binding curve between ALFA-tagged mNeonGreen and ALFA-tag binding nanobodies nbALFA and DnbALFA. f) SDS-PAGE analysis of expression of nbALFA and DnbALFA in 200 ml 2YT culture of *E.coli* BL21 (DE3) after single-step purification on Ni-NTA agarose. M – Protein ladder (kDa).

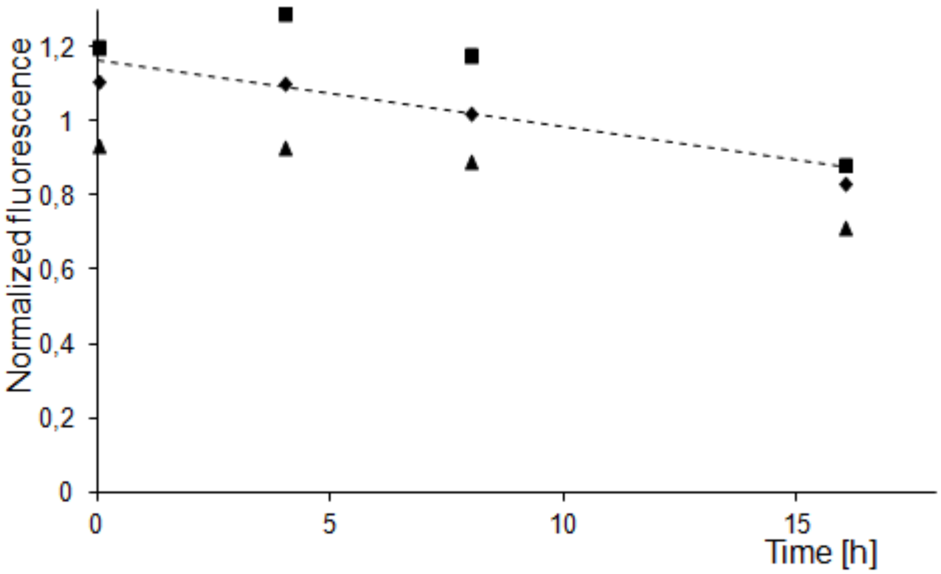

Figure S2 – The loss of fluorescence signal of ALFA tagged mNeonGreen in the conditioned media. The conditioned cell-free media was mixed with ALFA tagged mNeonGreen (20 nM) and its fluorescence was measured over time by Tecan infinite 200PRO plate reader (Tecan Life Sciences, Switzerland) with excitation 500 nm and emission 520 nM. The dashed line shows the average regression of three independent replicates.

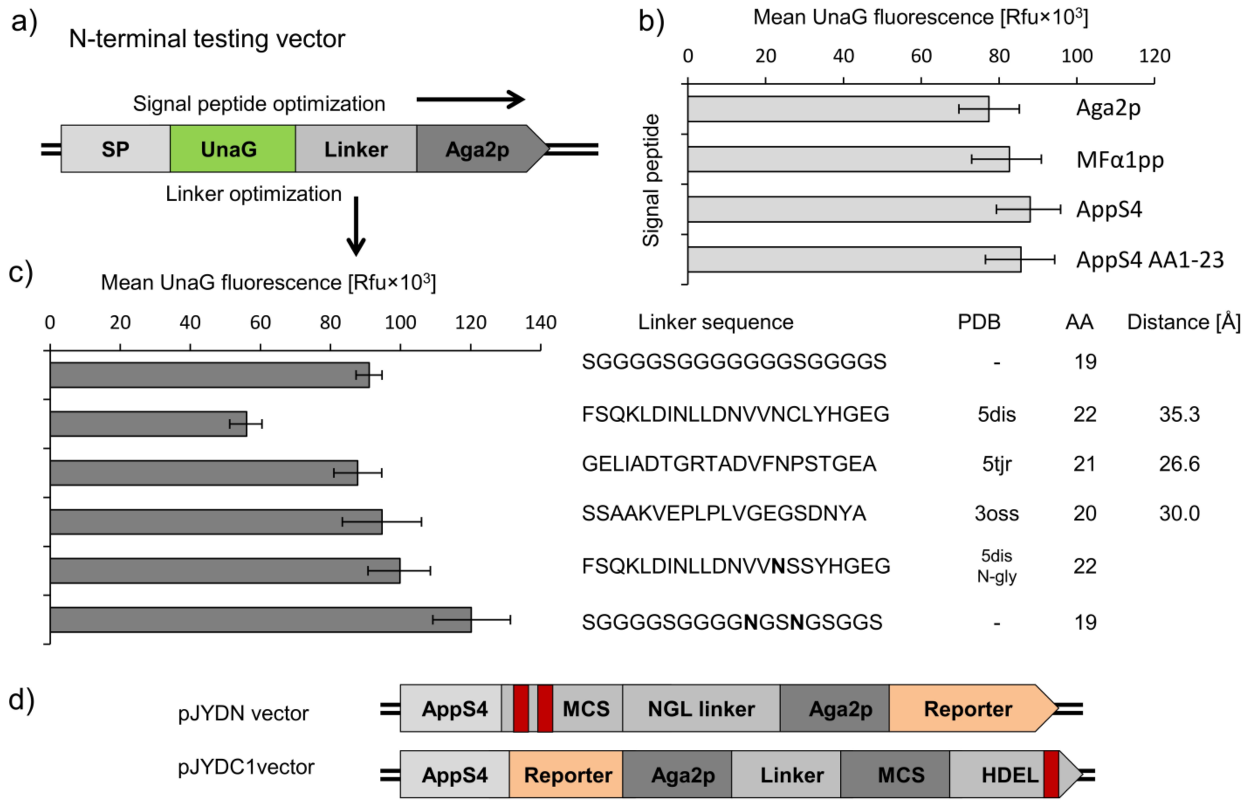

Figure S3 – Secretory leader and linkers optimization. a) Expression unit organization for the signal peptide and linkers optimizations for N-terminal fusion vectors. b) Cytometry based comparison of the impact of different signal peptides on UnaG expression at the N-terminus of Aga2p. The mean fluorescence values were recorded for 30 000 yeast cells per sample. c) The impact of different linkers between UnaG and Aga2p proteins on fluorescence intensity assessed by flow cytometry measurements. The asparagine residues in linker sequences, highlighted in bold, were introduced in order to gain N-glycosylations. Except for glycine-serine stretches, flexible linkers were isolated from regions not resolved in electron density maps of corresponding structures. d) Final organization of negative variants of N and C terminal vectors. Stop codons are highlighted by red stripes.

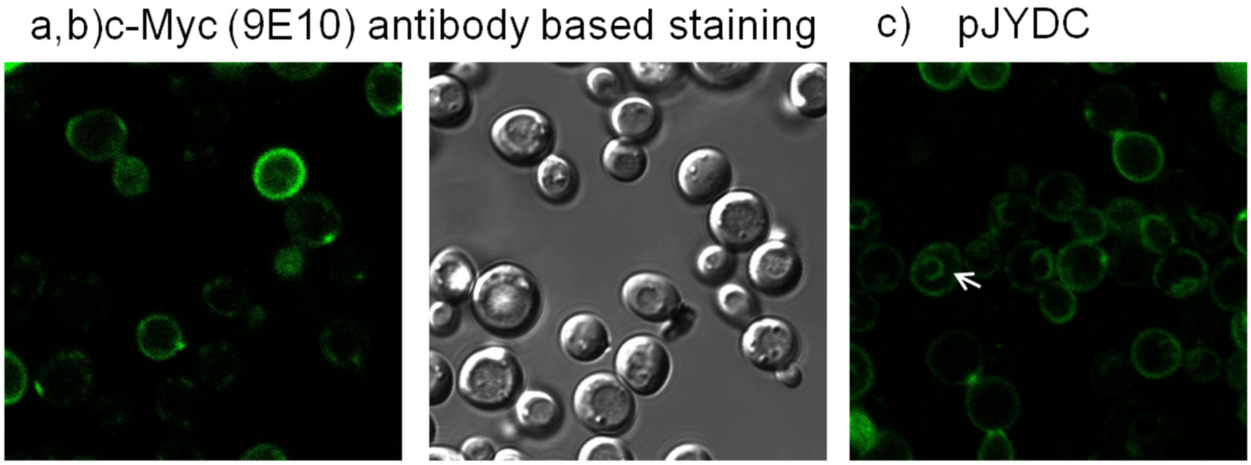

Figure S4 – pJYDC leaky expression analysis. a,b) The pJYDC1 expression analysis by using traditional c-myc labelling showed detectable surface expression from the empty plasmid - leaky expression of the pJYDC1 vector (media without bilirubin, HDEL sequence present). c) pJYDC1 expression analysis using eUnaG2 fluorescence signal (media with 5 nM bilirubin, white arrow highlights the endomembrane system signal). The empty pJYDC1 vector-based expression resulted in the low fluorescent signal, which was predominantly located in the endoplasmic reticulum due to the presence of the HDEL endoplasmic reticulum retention signal sequence.

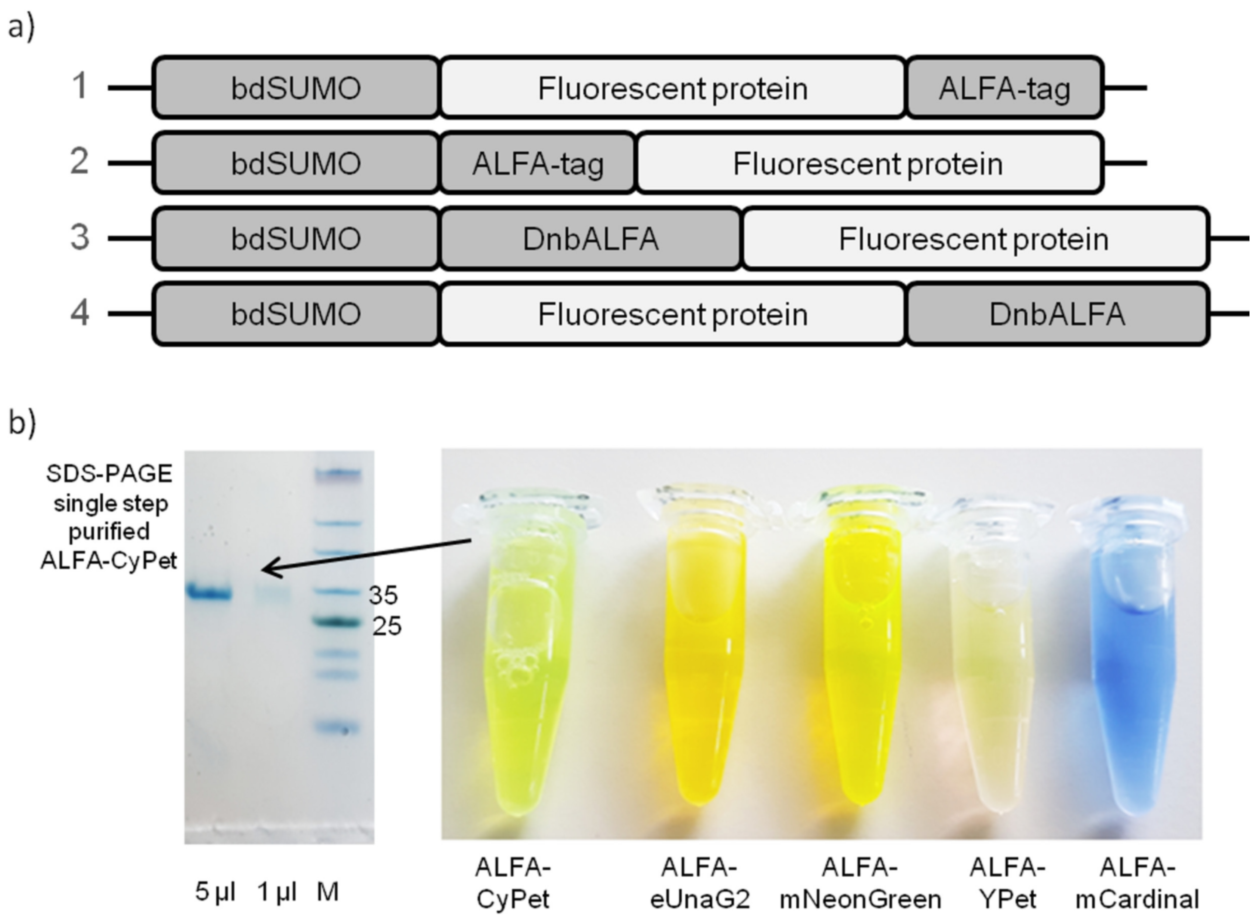

Figure S5 – ALFA-tagged fluorescent proteins and fusions with DnbALFA for yeast display. a) Schematic representation of tested protein fusion constructs. Constructs 1 and 4 show a substantially higher yield of soluble fusion protein than constructs 2 and 3. b) Single-step purified proteins for use in pJYD yeast display plasmids. Proteins were expressed in 600 ml (2YT media) culture of *E.coli* BL21 (DE3) and purified by bdSUMO on-column cleavage.

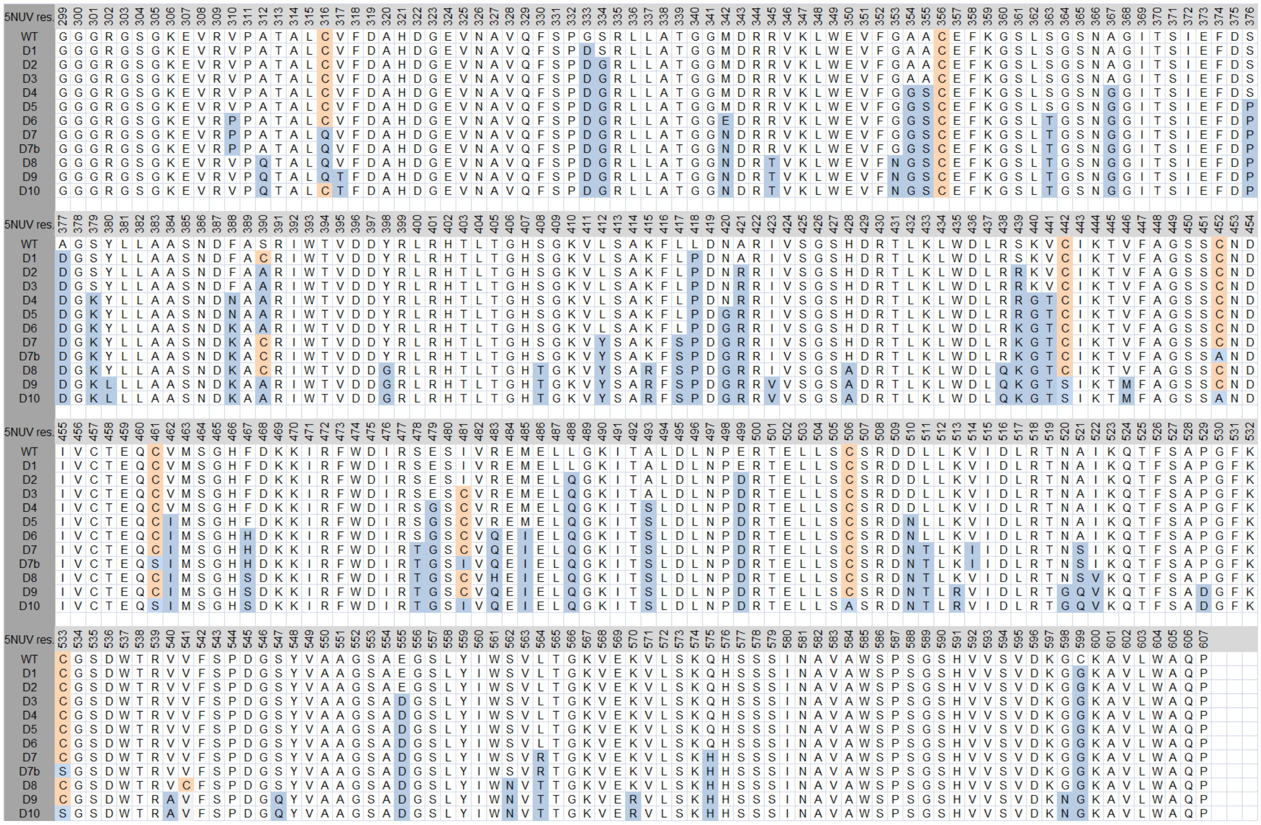

Figure S6 – Multiple sequence alignment of variants of WD40 domain of ATG16L1. The mutated residues except cysteines are highlighted by blue background. Cysteine residues are highlighted by orange background.

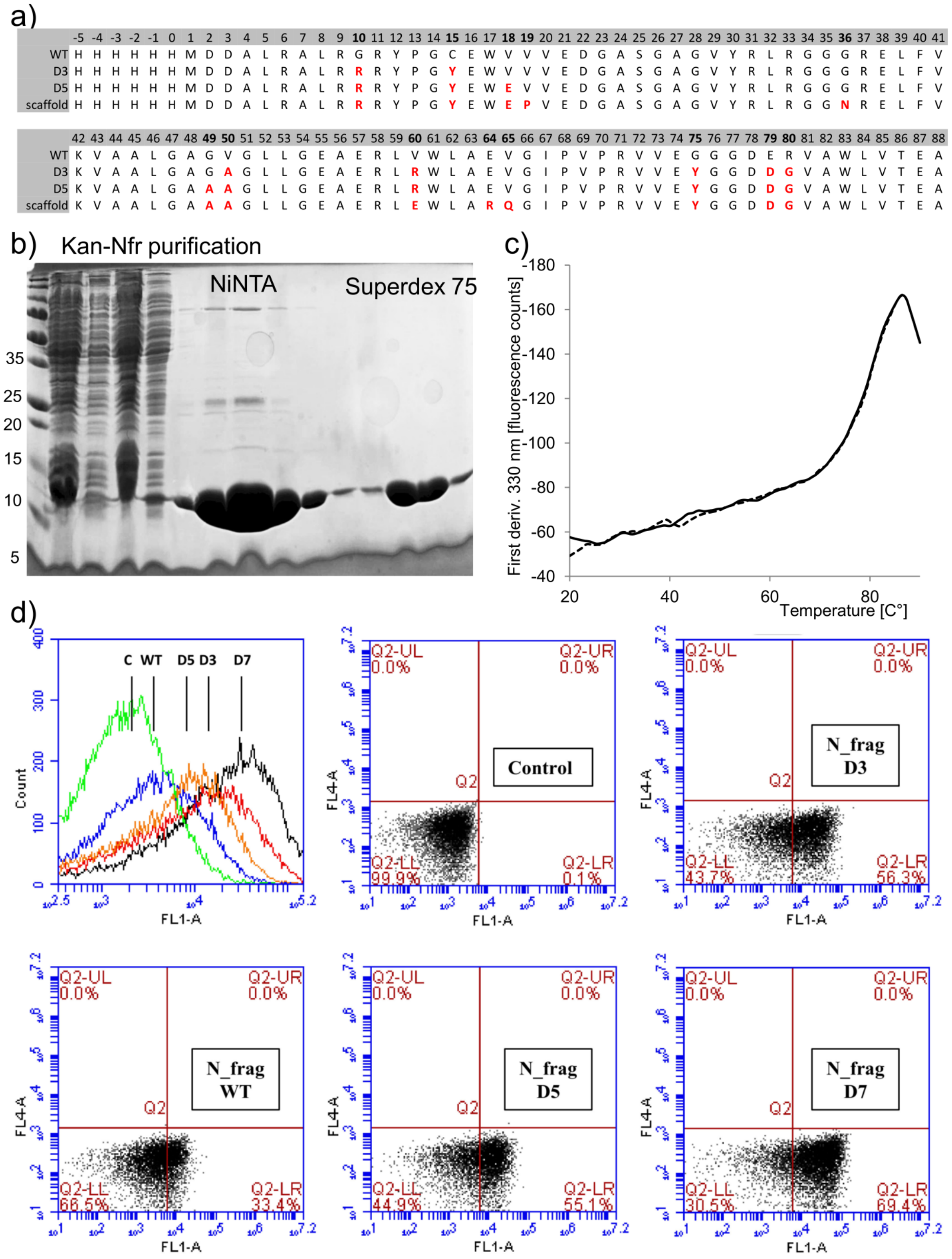

Figure S7 – Sequence design and testing of Kan-Nfr scaffold. a) Sequence alignment among wild type, design stabilized 4H05 N-fragment 3 (D3), design stabilized 4H05 N-fragment 5 (D5) and final design stabilized 4H05 N-fragment 7 (D7 or Kan-Nfr scaffold). Mutated residues are highlighted in red. b) SDS-PAGE analysis of Kan-Nfr scaffold expression in *E.coli* BL21(DE3) and its two-step purification on NiNTA agarose and Superdex 75 16/600 gel filtration chromatography. c) Melting of Kan-Nfr measured by using DSFnano Prometheus NT.48 instrument (Nanotemper Technologies, duplicate). d) *S.cerevisiae* EBY100 cell surface expression of wild type and stabilized 4H05 N-fragments analyzed by BD Accuri™ C6 Plus Flow Cytometer.

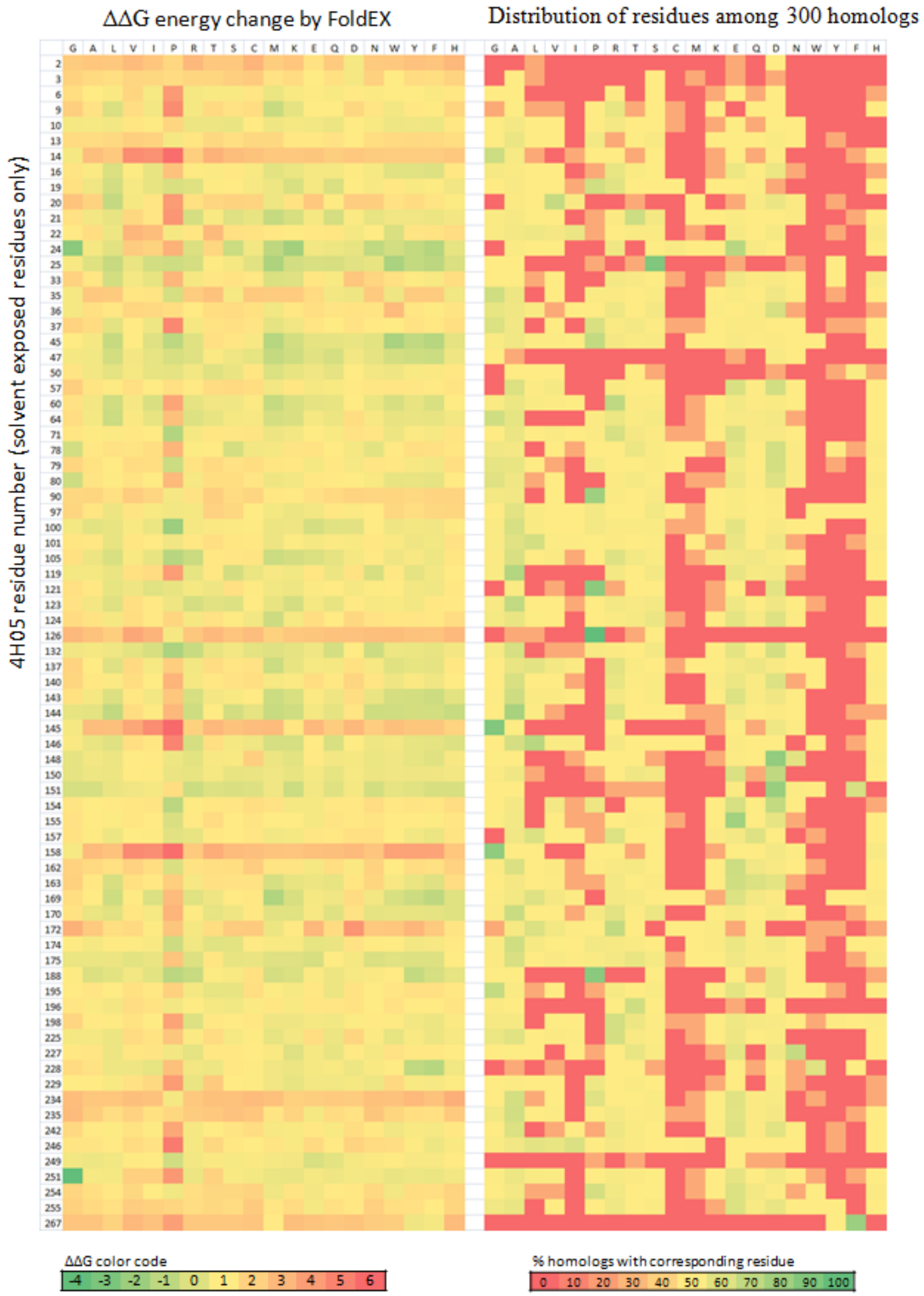

Figure S8 – FoldEX and MSA comparison for mutation site identification in PDB: 4H05 (full protein). Only amino-acid residues with more than 50% solvent accessibility were evaluated. Positions and patches in protein structure with large evolutionary variability and narrow energy scale were searched. Multiple libraries were proposed covering the N-terminal region (residues -5 – 92) and the C-terminal part (93 – 267).

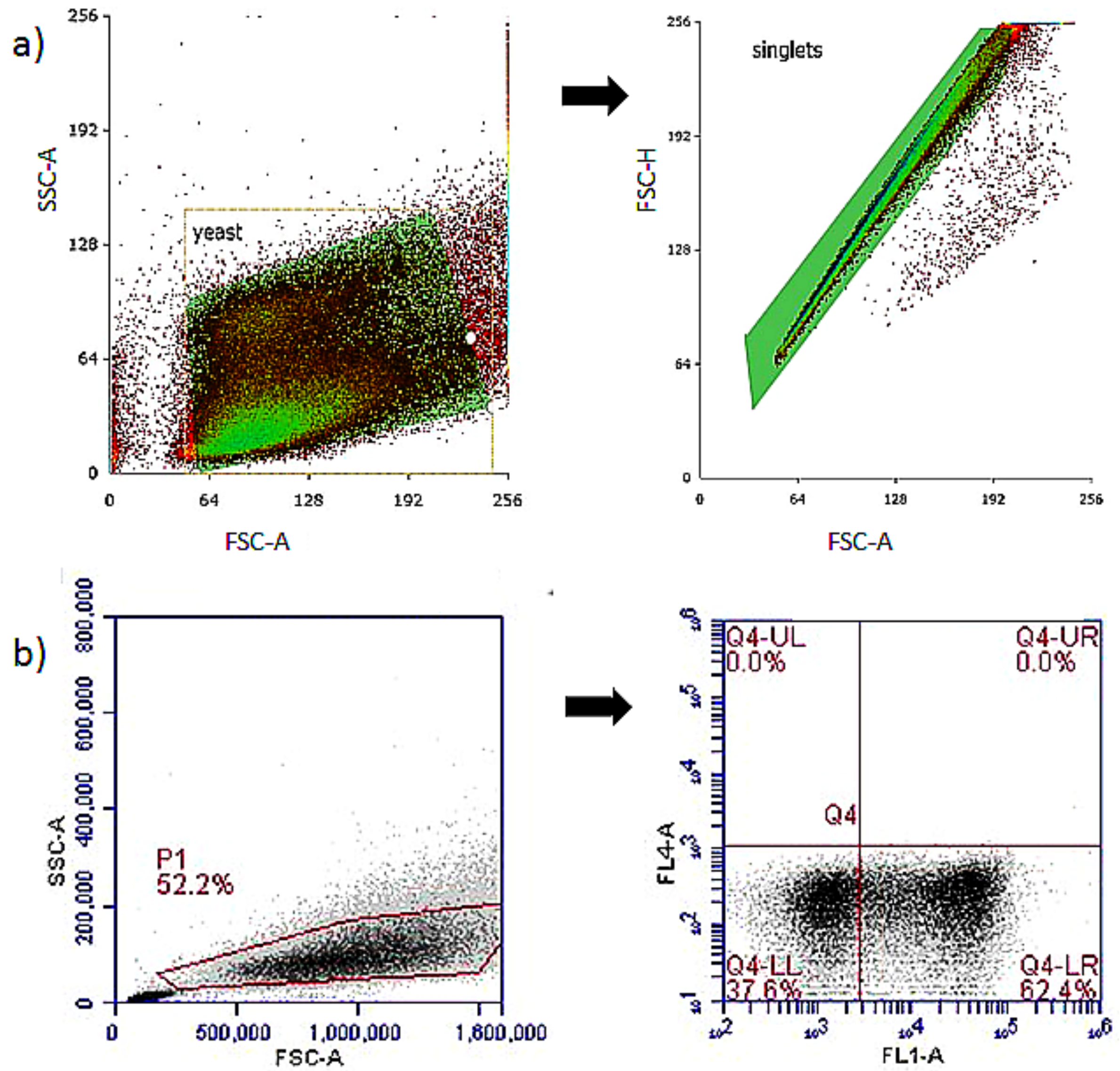

Figure S9. FACS gating strategy. a) Two-step gating strategy used in all sorting processes on S3e Cell Sorter device (Bio-Rad, USA). In the first step, yeast cells are isolated including mother and daughter populations. In the second gaiting, single cells are isolated by using FCS. b) Simplified single-step gating strategy used for analysis only on BD Accuri™ C6 Plus Flow Cytometer (BD Biosciences, USA). Quadrant gates were used to distinguish among negative cells (Q4-LL), cells positive for expression (eUnaG2 signal, Q4-LR), and binding/ double-positive cells (Q4-UR, not present in this example).

References used in Supplementary material

[1] Erijman A, Dantes A, Bernheim R, Shifman JM, Peleg Y. Transfer-PCR (TPCR): a highway for DNA cloning and protein engineering. J Struct Biol. 2011;175:171-7.
[2] Benatuil L, Perez JM, Belk J, Hsieh CM. An improved yeast transformation method for the generation of very large human antibody libraries. Protein engineering, design & selection : PEDS. 2010;23:155-9.
[3] Gietz RD, Woods RA. Transformation of yeast by lithium acetate/single-stranded carrier DNA/polyethylene glycol method. Methods in enzymology. 2002;350:87-96.
[4] Gietz RD, Schiestl RH. High-efficiency yeast transformation using the LiAc/SS carrier DNA/PEG method. Nature Protocols. 2007;2:31-4.
[5] Suga M, Isobe M, Hatakeyama T. Cryopreservation of competent intact yeast cells for efficient electroporation. Yeast. 2000;16:889-96.
[6] Zahradník J, Marciano S, Shemesh M, Zoler E, Chiaravalli J, Meyer B, et al. SARS-CoV-2 RBD evolution follows contagious mutation spread, yet generates an able infection inhibitor. bioRxiv. 2021:2021.01.06.425392.
[7] Chao G, Lau WL, Hackel BJ, Sazinsky SL, Lippow SM, Wittrup KD. Isolating and engineering human antibodies using yeast surface display. Nature Protocols. 2006;1:755-68.
[8] Cohen-Khait R, Schreiber G. Low-stringency selection of TEM1 for BLIP shows interface plasticity and selection for faster binders. Proceedings of the National Academy of Sciences of the United States of America. 2016;113:14982-7.
[9] Looke M, Kristjuhan K, Kristjuhan A. Extraction of genomic DNA from yeasts for PCR-based applications. Biotechniques. 2011;50:325-8.
[10] Bell J. A simple way to treat PCR products prior to sequencing using ExoSAP-IT. Biotechniques. 2008;44:834.
[11] Rakestraw JA, Sazinsky SL, Piatesi A, Antipov E, Wittrup KD. Directed evolution of a secretory leader for the improved expression of heterologous proteins and full-length antibodies in *Saccharomyces cerevisiae*. Biotechnol Bioeng. 2009;103:1192-201.
[12] Kjeldsen T, Pettersson AF, Hach M, Diers I, Havelund S, Hansen PH, et al. Synthetic leaders with potential BiP binding mediate high-yield secretion of correctly folded insulin precursors from *Saccharomyces cerevisiae*. Protein expression and purification. 1997;9:331-6.
[13] Schneider B, Gelly JC, de Brevern AG, Černý J. Local dynamics of proteins and DNA evaluated from crystallographic B factors. Acta crystallographica Section D, Biological crystallography. 2014;70:2413-9.
[14] Kumagai A, Ando R, Miyatake H, Greimel P, Kobayashi T, Hirabayashi Y, et al. A Bilirubin-Inducible Fluorescent Protein from Eel Muscle. Cell. 2013;153:1602-11.
